# Supplementary material for: First mobilisation after abdominal and cardiothoracic surgery: when is it actually performed? A national, multicentre, cross-sectional study
Source: BMJ Open. 2024 Feb 29;14(2):e082239. doi: 10.1136/bmjopen-2023-082239 (PMC10910679; doi:10.1136/bmjopen-2023-082239)
Supplement: Supplementary data [file bmjopen-2023-082239supp001.pdf]

Supplementary Table 1

The 18 hospitals included in the study.

**University Hospitals**<br>-Karolinska University Hospital, Stockholm<br>-Linköpings University Hospital<br>-Sahlgrenska University Hospital, Gothenburg<br>-Skånes University Hospital, Lund, and Malmö<br>-University Hospital of Umeå<br>-Uppsala Akademiska Hospital<br>-Örebro University Hospital<br><br>**Regional County Hospitals**<br>-Centrallasarettet, Växjö<br>-Danderyd hospital, Stockholm<br>-Halmstad hospital<br>-Norra Älvsborgs Länssjukhus, Trollhättan<br>-Södersjukhuset, Stockholm<br>-Västerås hospital<br>-Östersund hospital<br><br>**Local County Hospitals**<br>-Alingsås hospital<br>-Höglandssjukhuset, Eksjö<br>-Visby hospital<br>-Örnsköldsviks hospital

Fagevik Olsén M, *et al. BMJ Open* 2024; 14:e082239. doi: 10.1136/bmjopen-2023-082239
