# Supplementary figures and images for: First mobilisation after abdominal and cardiothoracic surgery: when is it actually performed? A national, multicentre, cross-sectional study
Source: BMJ Open. 2024 Feb 29;14(2):e082239. doi: 10.1136/bmjopen-2023-082239 (PMC10910679; doi:10.1136/bmjopen-2023-082239)

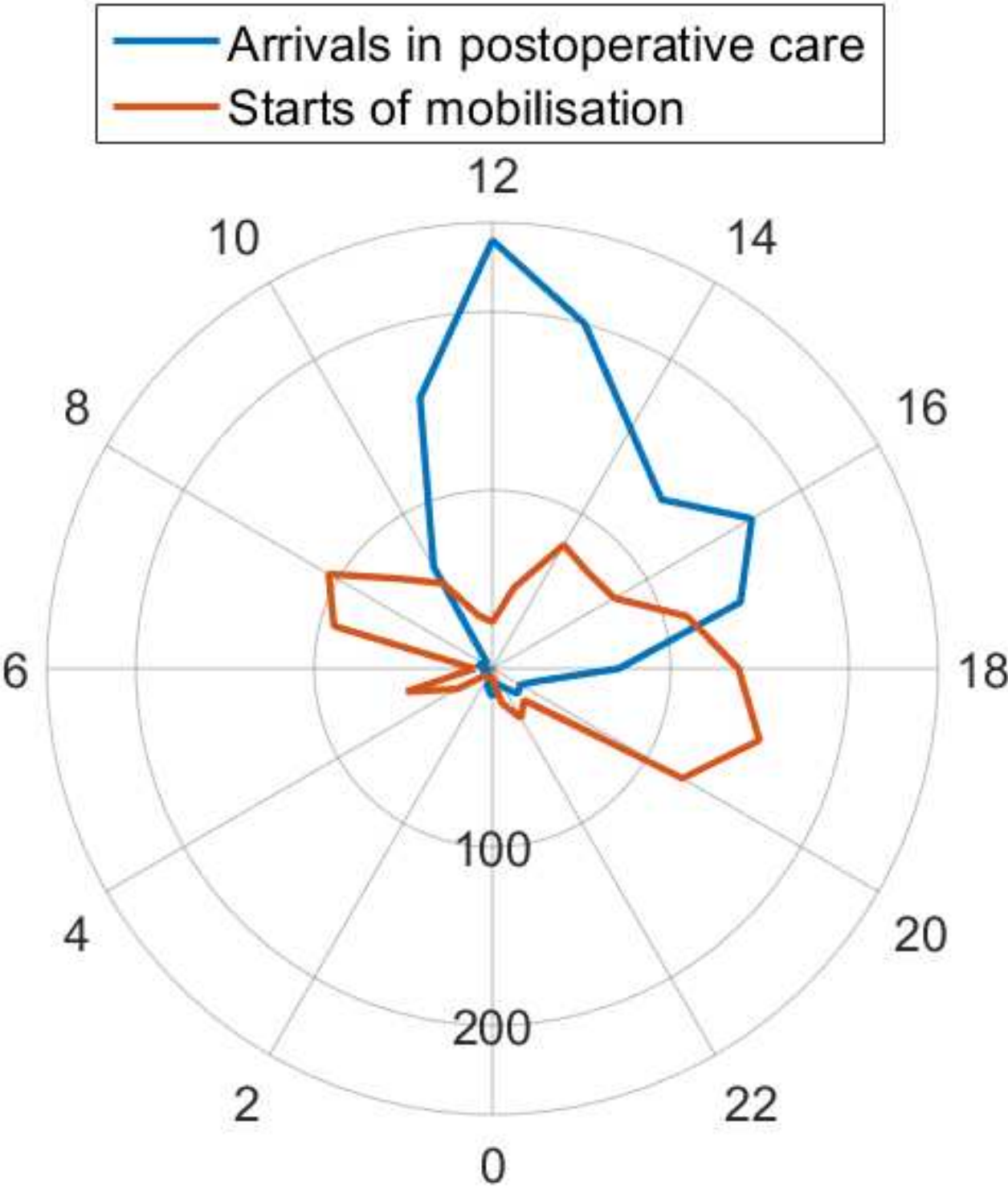

Supplement: Supplementary data [file bmjopen-2023-082239supp003.pdf]
