# Supplementary material for: First mobilisation after abdominal and cardiothoracic surgery: when is it actually performed? A national, multicentre, cross-sectional study
Source: BMJ Open. 2024 Feb 29;14(2):e082239. doi: 10.1136/bmjopen-2023-082239 (PMC10910679; doi:10.1136/bmjopen-2023-082239)
Supplement: Supplementary data [file bmjopen-2023-082239supp002.pdf]

Supplementary Table 2: Odds ratios for mobilisation within three and 12 hours after abdominal or cardiothoracic surgery.

Variable	Value	Mobilisation within 3 hours			Mobilisation within 12 hours		
		n (%) of event	OR (95%CI)	P-value	n (%) of event	OR (95%CI)	P-value
**Category of surgery**	Reference: Cardiac	56 (19.2%)	1.00		204 (69.9%)	1.00	<0.0001***
	Thoracic	17 (18.5%)	0.96 (0.52-1.74)	0.88	60 (65.2%)	0.81 (0.49-1.33)	0.40
	Major upper abd	9 (7.4%)	0.34 (0.16-0.70)	0.0038	58 (47.5%)	0.39 (0.25-0.60)	<0.0001
	Minor upper abd	83 (34.3%)	2.20 (1.48-3.26)	<0.0001	188 (78.0%)	1.53 (1.03-2.27)	0.034
	Intestinal	67 (20.2%)	1.07 (0.72-1.59)	0.74	229 (69.4%)	0.98 (0.69-1.38)	0.90
	Major Lower Abd	15 (16.9%)	0.85 (0.46-1.60)	0.62	59 (66.3%)	0.85 (0.51-1.41)	0.52
	Minor Lower Abd	32 (12.1%)	0.58 (0.36-0.93)	0.024	199 (75.4%)	1.32 (0.91-1.92)	0.15
**Procedure**	Reference: Elective	236 (21.1%)	1.00		817 (73.0%)	1.00	<0.0001***
	Acute ¤	21 (11.2%)	0.47 (0.29-0.76)	0.0019	100 (53.8%)	0.43 (0.31-0.59)	<0.0001
	Subacute ¤	19 (16.2%)	0.73 (0.43-1.21)	0.22	75 (64.1%)	0.66 (0.44-0.98)	0.042
**Duration of anaesthesia**	Reference: 0-4 h	150 (22.6%)	0.68 (0.52-0.89)	0.0047	522 (78.7%)	0.44 (0.35-0.56)	<0.0001
	>4-	126 (16.6%)			469 (62.0%)		
**Arrival in postoperative care**	Reference: Day	155 (23.5%)	1.00		551 (83.5%)	1.00	<0.0001***
	Evening	122 (17.8%)	0.70 (0.54-0.92)	0.0099	390 (56.9%)	0.26 (0.20-0.34)	<0.0001
	Night	2 (2.3%)	0.08 (0.02-0.32)	0.0004	56 (65.9%)	0.38 (0.23-0.63)	0.0001
**Perioperative bleeding, ml**	Reference: <999 ml	179 (17.5%)	0.31 (0.14-0.73)		710 (69.6%)	0.47 (0.31-0.72)	0.0005
	≥1000 ml	6 (6.3%)		0.0070	50 (52.1%)		
**Epidural anaesthesia**	Reference: No	244 (21.3%)	0.48 (0.32-0.72)	0.0003	820 (71.9%)	0.57 (0.43-0.75)	<0.0001
	Yes	31 (11.5%)			159 (59.1%)		
**Spinal anaesthesia**	Reference: No	256 (20.1%)	0.62 (0.38-1.01)	0.056	885 (69.7%)	0.90 (0.63-1.30)	0.59
	Yes	20 (13.5%)			100 (67.6%)		
**Sex**	Reference: Male	152 (20.1%)	0.93 (0.71-1.21)	0.58	510 (67.5%)	1.24 (0.99-1.56)	0.063
	Female	127 (18.9%)			483 (72.1%)		
**Age, years**	Reference: 18-69	176 (20.6%)	0.83 (0.64-1.09)	0.18	625 (73.3%)	0.66 (0.53-0.83)	0.0004
	70-	103 (17.8%)			372 (64.5%)		
**Obesity**	Reference: BMI<30	196 (18.6%)	1.22 (0.91-1.64)	0.18	723 (68.9%)	1.20 (0.92-1.57)	0.18
	BMI>30	78 (21.9%)			258 (72.7%)		
**Functional status**	Reference: Independant	278 (19.6%)	0.26 (0.03-1.94)	0.19	991 (70.1%)	0.26 (0.09-0.71)	0.0088
	Dependant	1 (5.9%)			6 (37.5%)		
**Received preoperative information**	Reference: No	171 (22.8%)	0.60 (0.46-0.79)	0.0002	499 (66.8%)	1.30 (1.03-1.63)	0.026
	Yes	100 (15.1%)			480 (72.3%)		
**Type of hospital**	Reference university	150 (15.9%)	1.00		622 (66.0%)	1.00	<0.0001***
	Regional county	104 (25.6%)	1.82 (1.37-2.41)	<0.0001	299 (74.0%)	1.47 (1.13-1.90)	0.0040
	Local county	25 (29.8%)	2.24 (1.36-3.69)	0.0016	76 (90.5%)	4.89 (2.33-10.25)	<0.0001
**Type of ward**	Reference: IMU	7 (18.4%)	1.00		26 (68.4%)	1.00	0.0079***
	Postop/ICU	227 (28.0%)	1.72 (0.75-3.97)	0.20	614 (75.8%)	1.45 (0.72-2.92)	0.30
	Surgical ward	42 (8.2%)	0.40 (0.16-0.96)	0.039	348 (68.1%)	0.99 (0.49-2.00)	0.97

Abd: Abdominal, ICU: Intensive Care Unit, IMU: Intermediate Unit, OR: Odds Ratio, Postop: Postoperative Unit. ***) p-value for the entire effect/factor/variable

¤ Acute surgery was defined as surgery within 24 hours after decision of surgery and subacute surgery within a week after the decision.

Fagevik Olsén M, *et al. BMJ Open* 2024; 14:e082239. doi: 10.1136/bmjopen-2023-082239
