# Supplementary material for: First mobilisation after abdominal and cardiothoracic surgery: when is it actually performed? A national, multicentre, cross-sectional study
Source: BMJ Open. 2024 Feb 29;14(2):e082239. doi: 10.1136/bmjopen-2023-082239 (PMC10910679; doi:10.1136/bmjopen-2023-082239)
Supplement: Author's manuscript [file bmjopen-2023-082239.draft_revisions.pdf]

BMJ Open

BMJ Open is committed to open peer review. As part of this commitment we make the peer review history of every article we publish publicly available.

When an article is published we post the peer reviewers' comments and the authors' responses online. We also post the versions of the paper that were used during peer review. These are the versions that the peer review comments apply to.

The versions of the paper that follow are the versions that were submitted during the peer review process. They are not the versions of record or the final published versions. They should not be cited or distributed as the published version of this manuscript.

BMJ Open is an open access journal and the full, final, typeset and author-corrected version of record of the manuscript is available on our site with no access controls, subscription charges or pay-per-view fees (http://bmjopen.bmj.com).

If you have any questions on BMJ Open's open peer review process please email info.bmjopen@bmj.com

First mobilisation after abdominal and cardiothoracic surgery - When is it actually performed? A national, multicentre, cross-sectional study

Journal:	*BMJ Open*
Manuscript ID	bmjopen-2023-082239
Article Type:	Original research
Date Submitted by the Author:	17-Nov-2023
Complete List of Authors:	Fagevik Olsén, Monika; Institute of Neuroscience and Physiology, Department of Health and Rehabilitation/Physiotherapy Department of Surgery; Sahlgrenska University Hospital, Department of Health and Rehabilitation/Physiotherapy Department of Surgery<br>Sehlin, Maria; Umeå University, Department of Community Medicine and Rehabilitation<br>Westerdahl, Elisabeth; Orebro universitet<br>Schandl, Anna; Karolinska Institute<br>Block, Linda; Gothenburg University, Department of Anaesthesiology and Intensive Care<br>Nygren-Bonnier, Malin; Medical Unit Occupational Therapy and Physiotherapy, Department of Neurobiology, Care Sciences and Society<br>Svensson-Raskh, Anna; Karolinska Institutet, Department of Neurobiology, Care Sciences and Society, Division of Physiotherapy; Karolinska University Hospital, Department of Allied Health Professionals, Functional Area Occupational Therapy and Physiotherapy
Keywords:	Cardiothoracic surgery < SURGERY, Physical Therapy Modalities, Colorectal surgery < SURGERY, Pancreatic surgery < SURGERY

SCHOLARONE™
Manuscripts

BMJ

I, the Submitting Author has the right to grant and does grant on behalf of all authors of the Work (as defined in the below author licence), an exclusive licence and/or a non-exclusive licence for contributions from authors who are: i) UK Crown employees; ii) where BMJ has agreed a CC-BY licence shall apply, and/or iii) in accordance with the terms applicable for US Federal Government officers or employees acting as part of their official duties; on a worldwide, perpetual, irrevocable, royalty-free basis to BMJ Publishing Group Ltd ("BMJ") its licensees and where the relevant Journal is co-owned by BMJ to the co-owners of the Journal, to publish the Work in this journal and any other BMJ products and to exploit all rights, as set out in our licence.

The Submitting Author accepts and understands that any supply made under these terms is made by BMJ to the Submitting Author unless you are acting as an employee on behalf of your employer or a postgraduate student of an affiliated institution which is paying any applicable article publishing charge ("APC") for Open Access articles. Where the Submitting Author wishes to make the Work available on an Open Access basis (and intends to pay the relevant APC), the terms of reuse of such Open Access shall be governed by a Creative Commons licence – details of these licences and which Creative Commons licence will apply to this Work are set out in our licence referred to above.

Other than as permitted in any relevant BMJ Author's Self Archiving Policies, I confirm this Work has not been accepted for publication elsewhere, is not being considered for publication elsewhere and does not duplicate material already published. I confirm all authors consent to publication of this Work and authorise the granting of this licence.

For peer review only

ORIGINAL MANUSCRIPT

First mobilisation after abdominal and cardiothoracic surgery - When is it actually performed?

A national, multicentre, cross-sectional study

Monika Fagevik Olsén, PhD[1], Maria Sehlin, PhD[2], Elisabeth Westerdahl, PhD[3], Anna Schandl, PhD[4], Linda Block, PhD[5], Malin Nygren-Bonnier, PhD[6], Anna Svensson-Raskh, PhD[6]

[1] Department of Health and Rehabilitation/Physiotherapy, Institute of Neuroscience and Physiology and Department of Surgery, Institute of Clinical Sciences, Sahlgrenska Academy, University of Gothenburg, Gothenburg, Sweden. [2] Department of Community, Medicine and Rehabilitation, Physiotherapy, Umeå University, Umeå, Sweden.[3]University Health Care Research Center and Department of Cardiothoracic and Vascular Surgery, Faculty of Medicine and Health, Örebro University, Örebro, Sweden. [4]Department of Molecular Medicine and Surgery, Karolinska Institutet, Stockholm, Sweden/ Department of Anaesthesiology and Intensive Care, Södersjukhuset, Stockholm, Sweden/ Department of Clinical Science and Education, Karolinska Institutet, Södersjukhuset, Sweden. [5]Department of Anaesthesiology and Intensive Care, Institute of Clinical Sciences, Sahlgrenska Academy, Gothenburg University, Gothenburg, Sweden/ Department of Anaesthesiology and Intensive Care, Sahlgrenska University Hospital, Gothenburg, Sweden. [6]Department of Neurobiology, Care Sciences and Society, Division of Physiotherapy, Karolinska Institutet, Stockholm, Sweden/ Women´s Health and Allied Health Professionals Theme, Medical Unit Occupational Therapy and Physiotherapy, Karolinska University Hospital, Stockholm, Sweden

Short title: Mobilisation after surgery

Word-count main text: 2626

Keywords: Abdominal Surgery, Cardiothoracic Surgery, Mobilisation, Postoperative.

Corresponding author:

Professor Monika Fagevik Olsén

Dept. of Physical Therapy,

Sahlgrenska Academy,

Gothenburg University,

SE 413 45 Gothenburg, Sweden

e-mail: monika.fagevik-olsen@gu.se

Abstract

Objectives: Knowledge of clinical practice regarding mobilisation after surgery is lacking.

This study therefore aimed to reveal current mobilisation routines after abdominal and cardiothoracic surgery and to identify factors associated with mobilisation within six hours postoperatively.

Design: A prospective observational national multicentre study

Setting: 18 different hospitals in Sweden

Participants: 1492 adult patients undergoing abdominal and cardiothoracic surgery with duration of anaesthesia >2 hours.

Primary and secondary outcomes: Primary outcome was time to first postoperative mobilisation. Secondary outcomes were the type and duration of the first mobilisation. Data were analysed using multivariate logistic regression and general structural equation modelling, and data are presented as odds ratios (ORs) with 95% confidence intervals (CIs).

Results: Among the included patients, 52% were mobilised to at lite bed edge sitting within six hours, 70% within 12 hours and 96% within 24 hours. Besides bed edge sitting, 76% stood up by the bed, and 22% were mobilised away from the bedside. Patients undergoing major upper abdominal surgery required the longest time before mobilisation with an average time of 11 hours post-surgery. Factors associated with increased likelihood of mobilisation within six hours of surgery were daytime arrival at the postoperative recovery unit (OR 5.19, 95%; CI 2.16-12.18), anaesthesia <4 hours (OR 1.68, 95%; CI 1.17-2.409, and ASA classification 1-2, (OR 1.63, 95%; CI 1.13-2.36).

Conclusions Most patients were mobilised within 24 hours after major surgery and 52% within six hours. Daytime arrival at the postoperative recovery unit, low ASA classification, and shorter duration of anaesthesia were associated with a shorter time to mobilisation.

For peer review only - http://bmjopen.bmj.com/site/about/guidelines.xhtml

Trial registrations: FoU in VGR (Id:275357) and Clinical Trials (NCT04729634).

Strengths and limitations of this study:

- The prospective, multicentre design of this study, including patients from all parts of Sweden, and the relatively high participation rate counteract selection bias.
- The large study sample enabled the planned analyses of the different surgical categories and exploration of related variables.
- Outcomes were determined by observations and relied on correct documentation by investigators.
- No patients were included during weekends, and therefore the results may not completely reflect common practice.
- Adverse events were not registered during the mobilisation. These would have given valuable information concerning the association between risk and when the mobilisation was performed.

Background

Mobilisation after abdominal and cardiothoracic surgery is an important intervention for postoperative recovery and prevention of complications, even though its specific effects are largely unexplored[1,2]. During decades of development pre, peri and postoperative care, it has been found that other active interventions such as re-inflation of the lungs at the end of anaesthesia may reduce the risk of postoperative pulmonary complications[3-6]. However, the specific effects of mobilisation are not yet known, nor how to facilitate it, nor when it should be initiated[7,8].

Undoubtedly, the change of position to sitting and/or standing has a multifactorial positive impact on cardiorespiratory function[8-10]. Lung volume increases with a subsequent decrease of the atelectic area, improving the ventilation/perfusion ratio and facilitating the removal of secretion[11-13]. The immediate respiratory effects of mobilisation have recently been evaluated in two randomised, controlled trials[14,15]. In those, mobilisation within the first hours after surgery was compared to immobilised control groups. The results indicate that changing position from supine to upright has advantages for cardiorespiratory function and no major adverse events of the intervention were reported.

Most hospitals claim that “early” mobilisation is implemented in their standard practice of care, but the term “early” is not clearly defined[7], and the current state of practice remains unknown. Therefore, the present study aimed to estimate the time of first mobilisation, and to identify factors associated with a mobilisation within six hours in a large cohort of adult patients who had undergone acute, sub-acute, or elective abdominal or cardiothoracic surgery.

For peer review only - http://bmjopen.bmj.com/site/about/guidelines.xhtml

Materials and methods

Design and setting

This is a part of the prospective observational national multicentre cohort study entitled "Survey Of Mobilisation and Breathing exercise After cardio-Thoracic and Abdominal surgery (SOMBATA)". Eighteen hospitals representing all parts of Sweden participated, including seven university hospitals and eleven county or local hospitals see supplementary file Table A. Data was collected between September 2021 and February 2022. The Swedish Ethical Review Authority approved the study (registration number 2020-03108) as well as all heads of the departments involved. Because of the observational study design of current practice, patient consent was waived. Patients were not involved in the design, or conduct, or reporting, or dissemination plans of our research.

Context of the study

In Sweden, most patients who have undergone abdominal and cardiothoracic surgery are monitored and supervised at intensive care units (ICUs), specific postoperative units or intermediate units (IMA) (a lower level of care than ICU, but a higher level of care than surgical wards), for some hours until they are considered to have sufficient circulatory and respiratory stability to be transferred to surgical wards. The standard care includes mobilisation within the first day after surgery, assisted by nurses, assistant nurses, and physiotherapists. Some hospitals included in this study followed multimodal perioperative care pathways for some of the categories of patients.

Inclusion

Adult (≥ 18 years) patients who had undergone open, laparoscopic, or robot-assisted surgery within the thorax or abdomen, with a duration of anaesthesia exceeding two hours were eligible for inclusion. Patients who had undergone plastic, trauma, transplant, or orthopaedic surgery or reoperation were excluded. Data was captured on patients undergoing surgery from Monday to Thursday.

All patients undergoing surgery were screened for participation by a responsible staff member at the study site.

Data collection

Patient characteristics and preoperative variables were retrieved from the patients' records. Data on perioperative variables, such as surgical procedure, duration of anaesthesia (in hours and minutes), blood loss (ml), and type of anaesthesia were obtained from the operation and postoperative surveillance charts. Type of surgery was categorised into: Cardiac (coronary artery bypass grafting, valve or aortic surgery via sternotomy); Thoracic (pulmonary surgery via thoracotomy or thoracoscopy); Major upper abdominal (pancreatic, hepatic, oesophageal and ventricular open procedures); Minor upper abdominal (open and laparoscopic procedures such as fundoplication and cholecystectomy); Intestinal (smaller intestine- and/or colorectal surgery); Major lower abdominal (extensive, open, gynaecological, urological, and other procedures with incision below the umbilicus); Minor lower abdominal (minor and laparoscopic gynaecological, urological, and other procedures with incision below the umbilicus).

The original research protocols were identifiable during the collection process to enable data completion from the patients´ records. When data was complete the protocols were de-identified before entering the results into a secure online database.

For peer review only - http://bmjopen.bmj.com/site/about/guidelines.xhtml

Outcomes

The primary outcome was time to first mobilisation from arrival at the postoperative unit. Mobilisation was defined as being able to at least sit on the edge of the bed. Secondary outcomes were the type and duration of the first mobilisation, number of staff assisting the mobilisation and furthermore to investigate which factors which were associated with an early mobilisation, defined as within six hours of surgery. Based upon the literature and clinical reasoning, the following data were considered potential factors influencing an early mobilisation, and were therefore collected; age, BMI, smoking, duration of anaesthesia, perioperative bleeding, co-morbidity (ASA classification), type of surgery, and arrival at the postoperative recovery unit[3,16-18].

The outcome data were registered, in parallel with the clinical work, in a case report form following the patient until the first mobilisation was completed or, if this did not happen, until 24 hours from admission at the postoperative unit. If no mobilisation was performed during the first 24 hours, the reason was noted in a bedside chart by the staff responsible for the trial at each study site.

Statistical Analysis

Continuous data were presented as means (standard deviation; s.d.) or median (min-max range), while categorical data were illustrated with numbers and percentages [%]. Differences between groups were analysed with ANOVA, Kruskal-Wallis test or Chi-square test.

The impact of baseline variables on the proportion of subjects and time to first mobilisation, time out of bed and type of mobilisation respectively, was analysed with univariable logistic regression. The significant univariable predictors were then entered stepwise into a multivariable logistic regression, using forward selection. Data were presented as odds ratios (ORs) with a 95% confidence interval (CI) and p-value.

For peer review only - http://bmjopen.bmj.com/site/about/guidelines.xhtml

A general structural equation model (GSEM) was applied to identify the associations between potential factors for being mobilised early and the time to first mobilisation, defined as within six hours from arrival at the postoperative unit[19]. A univariate logistic model identified relevant factors: age, sex, BMI, perioperative bleeding, smoking, duration of anaesthesia, ASA classification, time of arrival at the postoperative recovery unit and type of surgery. Thereafter a multivariate logistic regression was performed (including variables based on the statistical findings $p<0.05$), and to test for collinearity. The included variables were assessed stepwise and classified into the following categories: ASA classification (1-2 and 3-4)[11,17]; age (≤70 and >70 years)[3,11,18], arrival at the postoperative recovery unit (daytime/afternoon and evening/night-time)[11]; type of surgery (intestinal, minor upper and lower abdominal vs cardiac, thoracic, major upper and lower abdominal)[11,18]; and duration of anaesthesia (<4 and ≥4 hours)[3,11,18]. Finally, a GSEM was applied to analyse the structural association between the variables and mobilisation within six hours of arrival at the postoperative unit.

IBM SPSS version 29.0 and Stata Statistical Software: Release 17. College Station, TX, Stata Corp LLC were used for the analyses.

Results

Among 3802 patients who underwent abdominal or cardiothoracic surgery at the hospitals during the study periods, 1879 met the inclusion but not the exclusion criteria. Among them, 1492 patients (79%) were included in the study (Figure 1, Table 1).

For peer review only - http://bmjopen.bmj.com/site/about/guidelines.xhtml

Time to first mobilisation

Among the included patients, 279 (20%) were mobilised within three hours, 738 (52%) within six hours, 997 (70%) within 12 hours and 1396 (96%) within 24 hours. The mean time to the first mobilisation was 7:51 hours (sd 6:27) (Table 2). Major upper abdominal surgery was associated with the longest time to mobilisation (mean 11:03, sd 4:08 hrs) whereas minor upper abdominal procedures were associated with the shortest time (mean 5:07, sd 5:38 hrs). Supplementary figure shows the times that patients arrived at the postoperative care units during the day and night and when the first mobilisation was started. Most patients arrived at the postoperative wards between 10:30 a.m. and 5:00 p.m. Most of the first mobilisations started around 8 a.m. and between 2 and 8 p.m.

Fifty-nine patients (4%) were not mobilised within the first 24 hours of surgery, and stated reasons (several reasons could be listed) were: circulatory instability (n=7), respiratory instability (n=15), sedation (n=12), intubation (n=6), pain (n=9), nausea (n=2), tiredness (n=7) or not considered a priority by the health care professionals (n=3). The reasons were similar across seven categories of surgery (data not shown).

Time out of bed and type of mobilisation

The mean duration of time out of bed was 27 min (sd 1:16 hrs). Patients who had undergone cardiac surgery, intestinal surgery, and minor lower abdominal surgery had the shortest time out of bed during their first mobilisation (Table 2).

Besides that all patients who were mobilised sat on the bed edge, 76% stood up by the bed, 15% sat in a chair and 22% walked around the first time they were out of bed. There were significant differences between the groups of surgical categories in all activities ($p<0.001$). More patients who had undergone cardiac surgery stood up by the bed (89%), while more

patients who had undergone pulmonary surgery sat in a chair (82%) and/or walked around (18%) (P<0.001). Commonly, a nurse (60%) or an assistant nurse (60%) assisted with mobilisation (Table 2).

Factors associated with mobilisation.

The results of the odds ratio for mobilisation within six and 24 hours is presented in Table 3, and for three and 12 hours in the supplementary file. Minor upper abdominal surgery had the highest odds for mobilisation during the first six hours postoperatively. Decreased odds were factors as acute surgery, arrival at the postoperative care unit during evenings and nights, duration of anaesthesia >4 hours and bleeding >999 ml.

According to the results of the GSEM (Figure 3), the following of the included factors were found to increase the probability of being mobilised within six hours of surgery (conditional on other factors): an ASA classification of 1 and 2 (OR: 1.63; 95% CI: 1.13 to 2.36), arriving at the postoperative recovery unit in the daytime/afternoon (OR: 5.13; 95% CI: 2.16 to 12.18) and a duration of anaesthesia of less than four hours (OR: 1.68; 95% CI: 1.17 to 2.40).

For peer review only - http://bmjopen.bmj.com/site/about/guidelines.xhtml

Discussion

This is the first prospective study describing mobilisation routines after cardiothoracic and abdominal surgery. In this national, multicentre, observational study, which included a total of 1492 patients, it was found that most patients (96%), were mobilised within the first 24 hours, whereas more than half of the cohort (52%) were mobilised within six hours. In addition to sitting on the edge of the bed, 76% stood up during the first mobilisation. Arriving at the postoperative recovery unit in the daytime, a duration of anaesthesia of less than four hours, and having few co-morbidities (ASA 1 or 2) were independently associated with being mobilised within six hours of surgery.

Even though almost all patients were mobilised within 24 hours there were some differences regarding the time to first mobilisation between the different types of surgery. Possible explanations are different proportions of a malign cause of surgery, diverse methods of analgesia and, different inflammatory responses after the surgical trauma[20]. The duration of first mobilisation also differed between the surgery types but not to the same extent as the time to mobilisation. The average time out of bed was 27 min but was about twice that after lung surgery. A shorter duration of anaesthesia during the pulmonary procedures via thoracotomy may be one explanatory factor compared to cardiac surgery via sternotomy or larger abdominal incision with longer duration of anaesthesia. In addition, a previously published Danish study identified sternotomy and thoracoabdominal incision as clinically relevant risk factors for the development of postoperative pulmonary complications after major abdominal and cardiothoracic surgery[21].

A more upright position appears to be essential to normalise functional residual capacity and respiratory function after anaesthesia and surgery[22-24]. In addition, the upright position may also help to re-inflate the lung after atelectasis[12,13,25]. An earlier and more intensive mobilization, i.e., to standing and walking, may therefore be preferable, especially for those at

For peer review only - http://bmjopen.bmj.com/site/about/guidelines.xhtml

high risk of postoperative complications. In the current study, all mobilised patients sat on the edge of the bed, but a majority also stood up by the bed and 21 per cent even walked around. This is encouraging since the results from other studies show that compliance with Enhanced Recovery After Surgery (ERAS) recommendations is relatively poor with only 25-50% reaching the mobilisation targets[26,27]. Nevertheless, there are few absolute contraindications for mobilisation, and as including more active approaches in clinical pathways has been proven to reduce complications and accelerate recovery there may be room for improvement[7].

The findings of the present study indicate that arriving at the postoperative recovery unit in the daytime/afternoon is the strongest predictor of an earlier mobilisation as a low ASA classification and a duration of anaesthesia of less than four hours. This is in congruence with existing literature where factors such as advanced age, ASA classification ≥2, comorbidities, duration of anaesthesia ≥ 4 hours are associated with an increased risk for the development of postoperative pulmonary complications[11,17,18]. Since mobilisation is suggested to prevent postoperative pulmonary complications and has been found to be safe and feasible to perform for patients who have undergone advanced pulmonary[23], cardiac[8,12], abdominal[14,28], and pancreatic surgery[15], it may be reasonable to recommend targeting patients with higher ASA classification and longer duration of anaesthesia after major surgery. To facilitate mobilisation not only during the daytime, but it is also of great importance to have sufficient number of healthcare personnel who can assist in the procedures[29,30].

In 1958, Powels [31] suggested that "early mobilisation" should occur on the first day after operation and "prompt mobilisation" should occur on the day of surgery. However, surgery as well as pre-, peri- and postoperative care have developed considerably since then. Despite the frequent use of "early" there is still no consensus about its definition[2, 7]. In the current trial, national practice after different surgeries was investigated. The results indicate that more than half of the patients were mobilised within six hours. It remains to be evaluated whether

mobilisation within six hours after surgery may be used as a definition of early mobilisation in today's practice. But to increase transparency and avoid misuse and misinterpretation of the term "early mobilisation" it is high time to discuss and come to a consensus about a definition.

Strengths and limitations

The prospective, multicentre design of this study, including patients from all parts of Sweden, and the relatively high participation rate counteract selection bias. To determine the standard practice of postoperative mobilisation, we aimed to include at least 700 patients, leaving approximately 100 patients in each surgical category on which we could perform sub-group analyses. The large study sample enabled the planned analyses of the different surgical categories and exploration of related variables. Still, approximately 20% of the included patients had no registered outcome data. Outcomes were determined by observations and relied on correct documentation by investigators. There may be several reasons for the missing data, such as lack of staff, problems reaching all staff with information about the study, and logistic challenges. Another limitation was that no patients were included during weekends, and therefore the results may not completely reflect common practice. In addition, adverse events were not registered during the mobilisation. These would have given valuable information concerning the association between risk and when the mobilisation was performed.

Conclusion

Most of the patients in Sweden undergoing abdominal and cardiothoracic surgery that involved anaesthesia exceeding two hours were mobilised within the first 24 postoperative

hours, with more than half mobilised within six hours, and the first mobilisation included standing in the majority of cases. Factors associated with earlier mobilisation were daytime arrival at the postoperative recovery unit, shorter duration of anaesthesia, and few co-morbidities (low ASA classification). This overview of the national practice reveals unique baseline information to which other countries may be compared.

Funding statement: This study was financed by grants from the Swedish state under the agreement between the Swedish government and the county councils, the ALF-agreement (ALFGBG-965563), by The Local Research and Development Council, Gothenburg and Södra Bohuslän (VGFOUGSB-970106), and by Nyckelfonden, Örebro University Hospital Research Foundation.

Conflicts of interest: The authors declare no conflicts of interest.

Author contributions:

- MFO had full access to all the data in the study and was responsible for the integrity of the data and the accuracy of the data analysis.
- Concept and design: MFO, MS, EW, AS, LB, MNB, ASR
- Acquisition and analysis: MFO, ASR
- Interpretation of results: MFO, MS, EW, AS, LB, MNB, ASR
- Critical revision of the manuscript: MFO, MS, EW, AS, LB, MNB, ASR
- Statistical analysis: MFO, ASR and statistical consultants
- Guarantor: Gothenburg University

Data sharing statement*:* Data, Materials and Analytical Methods will be shared on request.

Figure legends:

Figure 1. Flow chart of the patients included in the study

Figure 2. General structural equation model (GSEM) analysis displaying correlations between time to first mobilisation, categorised as within six hours and the factors; ASA-classification; age; arrival at the postoperative recovery unit; type of surgery; duration of surgery.

Supplementary Figure . Number of participants arriving in postoperative care and number of participants starting mobilisation during the night and day.

For peer review only - http://bmjopen.bmj.com/site/about/guidelines.xhtml

References

1 Ljungqvist O, Scott M, Fearon KC. Enhanced Recovery After Surgery: A Review. JAMA Surgery 2017;152: 292-8

2 Tazreean R, Nelson G, Twomey R. Early mobilization in enhanced recovery after surgery pathways: current evidence and recent advancements. J Comp Eff Res 2022;11: 121-9

3 Gallart L, Canet J. Post-operative pulmonary complications: Understanding definitions and risk assessment. Best practice & research Clinical anaesthesiology 2015;29: 315-30

4 Westerdahl E, Lindmark B, Eriksson T, et al. Deep-breathing exercises reduce atelectasis and improve pulmonary function after coronary artery bypass surgery. Chest 2005;128: 3482-8

5 Yang D, Grant MC, Stone A, et al. A Meta-analysis of Intraoperative Ventilation Strategies to Prevent Pulmonary Complications: Is Low Tidal Volume Alone Sufficient to Protect Healthy Lungs? Ann Surg 2016;263: 881-7

6 Odor PM, Bampoe S, Gilhooly D, et al. Perioperative interventions for prevention of postoperative pulmonary complications: systematic review and meta-analysis. BMJ 2020;368: m540

7 Castelino T, Fiore JF, Jr., Niculiseanu P, et al. The effect of early mobilization protocols on postoperative outcomes following abdominal and thoracic surgery: A systematic review. Surgery 2016;159: 991-1003

8 Ramos Dos Santos PM, Aquaroni Ricci N, et al. Effects of early mobilisation in patients after cardiac surgery: a systematic review. Physiotherapy 2017;103: 1-12.

9 Hsu HO, Hickey RF. Effect of posture on functional residual capacity postoperatively. Anesthesiology 1976;44: 520-1

10 Katz S, Arish N, Rokach A, et al. The effect of body position on pulmonary function: a systematic review. BMC Pulm Med 2018;18: 159

11 Miskovic A, Lumb AB. Postoperative pulmonary complications. Br J Anaesth 2017;118: 317-34

12 Moradian ST, Najafloo M, Mahmoudi H, Ghiasi MS. Early mobilization reduces the atelectasis and pleural effusion in patients undergoing coronary artery bypass graft surgery: A randomized clinical trial. J Vasc Nurs 2017;35: 141-5

13 Hanada M, Kanetaka K, Hidaka S, et al. Effect of early mobilization on postoperative pulmonary complications in patients undergoing video-assisted thoracoscopic surgery on the esophagus. Esophagus 2018;15: 69-74

14 Svensson-Raskh A, Schandl AR, Ståhle A, et al. Mobilization Started Within 2 Hours After Abdominal Surgery Improves Peripheral and Arterial Oxygenation: A Single-Center Randomized Controlled Trial. Phys Ther 2021;101

15 Fagevik Olsén M, Becovic S, Dean E. Short-term effects of mobilization on oxygenation in patients after open surgery for pancreatic cancer: a randomized controlled trial. BMC Surgery 2021;21: 185

16 Fernandez-Bustamante A, Frendl G, Sprung J, et al. Postoperative Pulmonary Complications, Early Mortality, and Hospital Stay Following Noncardiothoracic Surgery: A Multicenter Study by the Perioperative Research Network Investigators. JAMA Surg 2017;152: 157-66

17 Smetana GW, Lawrence VA, Cornell JE. Preoperative pulmonary risk stratification for noncardiothoracic surgery: systematic review for the American College of Physicians. Ann Inter Med 2006;144: 581-95

18 Neto AS, da Costa LGV, Hemmes SNT, et al. The LAS VEGAS risk score for prediction of postoperative pulmonary complications: An observational study. Eur J Anaesthesiol 2018;35: 691-701

For peer review only - http://bmjopen.bmj.com/site/about/guidelines.xhtml

19 Armstrong RA. Recommendations for analysis of repeated-measures designs: testing and correcting for sphericity and use of manova and mixed model analysis. Ophthalmic and Physiological Optics. 2017;37: 585-93

20 Giannoudis PV, Dinopoulos H, Chalidis B, Hall GM. Surgical stress response. 2006;37 Suppl 5: S3-9

21 Salling SL, Jensen JH, Mosegaard SB, et al. Risk stratification for post-operative pulmonary complications following major cardiothoracic or abdominal surgery: Validation of the PPC Risk Prediction Score for physiotherapist's clinical decision-making. CRJ 2023;17: 229-40

22 Nielsen KG, Holte K, Kehlet H. Effects of posture on postoperative pulmonary function. Acta Anaesthesiol Scand 2003;47: 1270-5

23 Kaneda H, Saito Y, Okamoto M, et al. Early postoperative mobilization with walking at 4 hours after lobectomy in lung cancer patients. Gen Thorac Cardiovasc Surg 2007;55: 493-8

24 Mynster T, Jensen LM, Jensen FG, et al. The effect of posture on late postoperative oxygenation. Anaesthesia 1996;51: 225-7

25 Lindberg P, Gunnarsson L, Tokics L, et al. Atelectasis and lung function in the postoperative period. Acta Anaesthesiol Scand 1992;36: 546-53

26 Grass F, Pache B, Martin D, et al. Feasibility of early postoperative mobilisation after colorectal surgery: A retrospective cohort study. Int J Surgery 2018;56: 161-6

27 Gustafsson UO, Hausel J, Thorell A, et al. Adherence to the enhanced recovery after surgery protocol and outcomes after colorectal cancer surgery. Arch Surg 2011;146: 571-7

28 Thörn RW, Stepniewski J, Hjelmqvist H, et al. Supervised Immediate Postoperative Mobilization After Elective Colorectal Surgery: A Feasibility Study. World J Surg 2022;46: 34-42

29 Garzon-Serrano J, Ryan C, Waak K, et al. Early mobilization in critically ill patients: patients' mobilization level depends on health care provider's profession. PM & R 2011;3: 307-13

30 Fletcher N, Engelman DT. Postoperative care: who should look after patients following surgery? Anaesthesia.2020;75: e5-e9

31 Powers JH. Early ambulation: its influence on postoperative complications and return to work following hernioplasty in a rural population. Ann N Y Acad Sci 1958;73: 524-36

For peer review only - http://bmjopen.bmj.com/site/about/guidelines.xhtml

Table 1. Demographic data of the included patients. Data presented as mean (sd) or n [%].

		All, n=1492	Cardiac, n=292	Thoracic n=94	Major upper abd, n=126	Minor upper abd, n=257	Intestinal n=347	Major lower abd n=94	Minor lower abd n=282
Preoperative factors									
Age, years		62.2 (16.0)	63.5 (13.7)	60.4 (17.6)	66.6 (12.7)	56.0 (18.5)	67.1 (15.6)	62.5 (13.7)	59.3 (15.3)
Male sex, n		784 (49.1%)	227 (77.7%)	49 (52.1%)	81 (64.3%)	96 (37.4%)	175 (50.4%)	33 (35.1%)	123 (43.6%)
BMI, kg/m^2		27.7 (5.5)	27.0 (4.9)	25.7 (4.5)	27.0 (4.8)	29.1 (6.4)	26.3 (5.8)	26.4 (5.1)	27.0 (5.3)
Functional status- partly/totally dependent, n		18 (1.1%)	2 (0.7%)	0	1 (0.8%)	5 (1.9%)	9 (2.6%)	0	1 (0.4%)
ASA, score	1	171 (11.8%)	0	6 (6.4%)	8 (6.8%)	42 (17.1%)	30 (8.8%)	14 (14.9%)	71 (25.5%)
	2	575 (39.5%)	12 (4.2%)	37 (39.4%)	53 (44.9%)	119 (48.4%)	156 (46.0%)	641 (68.1%)	134 (48.2%)
	3	611 (42.0%)	212 (74.4%)	48 (51.1%)	56 (47.5%)	73 (28.4%)	136 (40.1%)	15 (16.0%)	71 (25.5%)
	4	96 (6.6%)	60 (21.1%)	3 (3.2%)	1 (0.8%)	12 (4.7%)	17 (5.0%)	1 (1.1%)	2 (0.7%)
	5	1 (0.1%)	1 (0.4%)	0	0	0	0	0	0
	Missing	38	7	0	8	11	8	0	4
Perioperative factors									
Duration of anaesthesia, h		4.60 (2.17)	4.80 (1.46)	3.66 (1.12)	7.22 (3.12)	3.43 (1.35)	5.26 (2.45)	4.72 (1.81)	3.77 (1.41)
Duration of surgery, h		3.27 (2.16)	3.41 (1.95)	2.03 (1.07)	5.53 (2.98)	2.33 (1.20)	3.90 (2.29)	3.34 (1.73)	2.59 (1.74)
Type of surgery, n	Open	826 (55.6%)	282 (96.9%)	31 (33.0%)	102 (81.6%)	68 (26.7%)	195 (53.5%)	60 (63.8%)	88 (31.3%)

For peer review only - http://bmjopen.bmj.com/site/about/guidelines.xhtml

Table 2. Time to first mobilisation, duration, and type of mobilisation, presented as mean (s.d.), median (Q1:Q3) or n [per cent, %].

		All, n=1492	Cardiac, n=292	Thoracic n=94	Major upper abd n=126	Minor upper abd, n=257	Intestinal n=347	Major lower abd n=94	Minor lower abd n=282	P value
Time to first mobilisation, h		7.91 (5.93) 5.50 (3.30:13.02)	8.20 (5.47) 6.67 (4.00:12.50)	8.39 (6.61) 4.92 (3.31:15.06)	11.05 (5.80) 11.80 (4.84:16.29)	5.95 (5.63) 3.75 (2.22:7.41)	7.48 (8.02) 5.25 (3.25:13.72)	8.75 (6.21) 6.10 (3.31:15.70)	7.63 (5.68) 2.26 (3.92:8.71)	<.001
Time to first mobilisation, categorised in intervals, hrs	<3	279 (20.4%)	56 (20.1%)	15 (16.9%)	11 (9.3%)	83 (36.6%)	67 (21.6%)	15 (17.0%)	32 (12.4%)	<0.001
	3-<6	458 (33.4%)	64 (22.9%)	39 (44.0%)	25 (21.2%)	76 (33.5%)	111 (35.8%)	28 (31.8%)	115 (44.4%)	
	6-<9	186 (13.6%)	55 (19.7%)	4 (4.5%)	9 (7.6%)	18 (7.9%)	36 (11.6%)	14 (15.9%)	50 (19.3%)	
	9-<12	74 (5.4%)	29 (10.4%)	5 (5.6%)	12 (10.2%)	9 (4.0%)	15 (4.8%)	2 (2.2%)	2 (0.8%)	
	12-<15	103 (7.5%)	30 (10.8%)	8 (9.0%)	20 (16.9%)	11 (4.8%)	18 (5.8%)	3 (3.4%)	13 (5.0%)	
	15-<18	160 (11.7%)	30 (10.8%)	10 (11.2%)	28 (23.9%)	15 (6.6%)	35 (11.3%)	18 (20.5%)	24 (9.3%)	
	18-<21	87 (6.4%)	15 (5.4%)	3 (3.4%)	11 (9.3%)	12 (5.3%)	22 (7.1%)	7 (8.0%)	17 (6.6%)	
	21-24	23 (1.8%)	0	5 (5.6%)	2 (1.8%)	3 (1.3%)	6 (1.8%)	1	6 (2.3%)	
	No time reg.	26	0	2	2	6	7	3	6	
	Not mobilised	62	13	2	5	15	22	1	4	
	Missing	34	0	1	1	9	8	2	13	
Mobilised within 24 hrs, n	Not mobilised	62 (4.2%)	13 (4.1%)	2 (2.2%)	5 (4.0%)	15 (6.0%)	19 (5.5%)	1 (1.1%)	4 (1.5%)	.071
	Mobilised	1430 (95.8%)	279 (95.9%)	91 (97.8%)	120 (96.0%)	233 (94.0%)	317 (94.3%)	91 (98.9%)	265 (98.5%)	
Duration of mobilisation, h		0.45 (1.27) 0.17 (0.17:0.33)	0.34 (0.68) 1.67 (0.08:0.25)	0.96 (2.55) 0.50 (0.17:1.00)	0.49 (0.69) 0.25 (0.17:0.50)	0.58 (1.82) 0.25 (0.17:0.48)	0.31 (0.41) 0.67 (0.67:0.33)	0.27 (0.29) 0.67 (0.12:0.30)	0.47 (1.56) 0.17 (0.08-0.33)	<.001

Table 3. Odds ratios for mobilisation within six and 24 hours after abdominal or cardiothoracic surgery.

Variable	Value	Mobilisation within 6 hours			Mobilisation within 24 hours		
		n (%) of event	OR (95%CI)	P-value	n (%) of event	OR (95%CI)	P-value
**Category of surgery**	Reference: Cardiac	120 (41.1%)	1.00	<0.0001***	279 (95.5%)	1.00	0.12***
	Thoracic	55 (59.8%)	2.13 (1.32-3.43)	0.0019	91 (97.8%)	2.12 (0.47-9.57)	0.33
	Major upper abd	34 (27.9%)	0.55 (0.35-0.88)	0.012	118 (95.2%)	0.92 (0.34-2.47)	0.86
	Minor upper abd	160 (66.1%)	2.80 (1.96-3.98)	<0.0001	234 (94.4%)	0.78 (0.36-1.69)	0.53
	Intestinal	179 (54.1%)	1.69 (1.23-2.32)	0.0012	317 (94.3%)	0.78 (0.38-1.60)	0.50
	Major Lower Abd	43 (48.3%)	1.34 (0.83-2.16)	0.23	91 (98.9%)	4.24 (0.55-32.86)	0.17
	Minor Lower Abd	147 (55.7%)	1.80 (1.29-2.52)	0.0006	265 (98.5%)	3.09 (0.99-9.59)	0.051
**Procedure**	Reference: Elective	620 (55.4%)	1.00	<0.0001***	1119 (98.5%)	1.00	<0.0001***
	Acute	62 (33.0%)	0.40 (0.29-0.55)	<0.0001	155 (81.6%)	0.07 (0.04-0.12)	<0.0001
	Subacute	52 (44.4%)	0.64 (0.44-0.94)	0.024	112 (94.1%)	0.24 (0.10-0.60)	0.0021
**Duration of anaesthesia**	Reference: 0-4 h	385 (58.0%)	0.62 (0.50-0.76)	<0.0001	651 (96.7%)	0.71 (0.41-1.22)	0.22
	>4-	349 (46.0%)			736 (95.5%)		
**Arrival in postoperative care**	Reference: Day	404 (61.2%)	1.00	<0.0001***	652 (97.8%)	1.00	<0.0001***
	Evening	323 (47.1%)	0.56 (0.45-0.70)	<0.0001	676 (96.8%)	0.71 (0.36-1.37)	0.31
	Night	11 (12.8%)	0.09 (0.05-0.18)	<0.0001	67 (75.3%)	0.07 (0.03-0.14)	<0.0001
**Perioperative bleeding, ml**	Reference: <999 ml	509 (49.9%)	0.48 (0.31-0.75)		999 (96.3%)	0.58 (0.24-1.40)	0.22
	≥1000 ml	31 (32.3%)		0.0012	91 (93.8%)		
**Epidural anaesthesia**	Reference: No	606 (53.0%)	0.68 (0.52-0.89)	0.0051	1115 (96.0%)	0.82 (0.44-1.54)	0.54
	Yes	117 (43.5%)			259 (95.2%)		
**Spinal anaesthesia**	Reference: No	653 (51.4%)	0.97 (0.69-1.37)	0.87	1233 (95.7%)	2.24 (0.69-7.25)	0.18
	Yes	75 (50.7%)			148 (98.0%)		
**Sex**	Reference: Male	373 (49.3%)	1.20 (0.97-1.47)	0.093	732 (95.6%)	1.22 (0.72-2.07)	0.45
	Female	361 (53.8%)			658 (96.3%)		
**Age, years**	Reference: 18-69	461 (54.0%)	0.78 (0.63-0.96)	0.021	842 (97.1%)	0.48 (0.28-0.82)	0.0068

For peer review only - http://bmjopen.bmj.com/site/about/guidelines.xhtml

Variable	Value	Mobilisation within 6 hours			Mobilisation within 24 hours		
		n (%) of event	OR (95%CI)	P-value	n (%) of event	OR (95%CI)	P-value
	70-	277 (47.8%)			553 (94.2%)		
**Obesity**	Reference: BMI<30	534 (50.8%)	1.17 (0.92-1.49)	0.20	1026 (96.2%)	1.14 (0.59-2.20)	0.69
	BMI>30	195 (54.8%)			351 (96.7%)		
**Functional status**	Reference: Independant	735 (51.9%)	1.00		1382 (96.2%)	0.10 (0.03-0.30)	<0.0001
	Dependant	3 (17.6%)	0.20 (0.06-0.69)	0.011	13 (72.2%)		
**Received preoperative information**	Reference: No	392 (52.3%)	0.90 (0.73-1.11)	0.32	715 (93.7%)	4.03 (2.08-7.83)	<0.0001
	Yes	330 (49.7%)			661 (98.4%)		
**Type of hospital**	Reference university	448 (47.6%)	1.00	<0.0001***	919 (96.0%)	1.00	0.33***
	Regional county	232 (57.1%)	1.47 (1.16-1.86)	0.0013	393 (95.2%)	0.81 (0.47-1.41)	0.46
	Local county	58 (69.0%)	2.46 (1.52-3.97)	0.0002	83 (98.8%)	3.43 (0.47-25.31)	0.23
**Type of ward**	Reference: IMU	23 (60.5%)	1.00	0.0005***			
	Postop/ICU	468 (57.8%)	0.89 (0.46-1.74)	0.74			
	Surgical ward	240 (47.0%)	0.58 (0.29-1.13)	0.11			

Abd: Abdominal, ICU: Intensive Care Unit, IMU: Intermediate Unit, OR: Odds Ratio, Postop: Postoperative Unit.

***) P-value for the entire effect/factor/variable

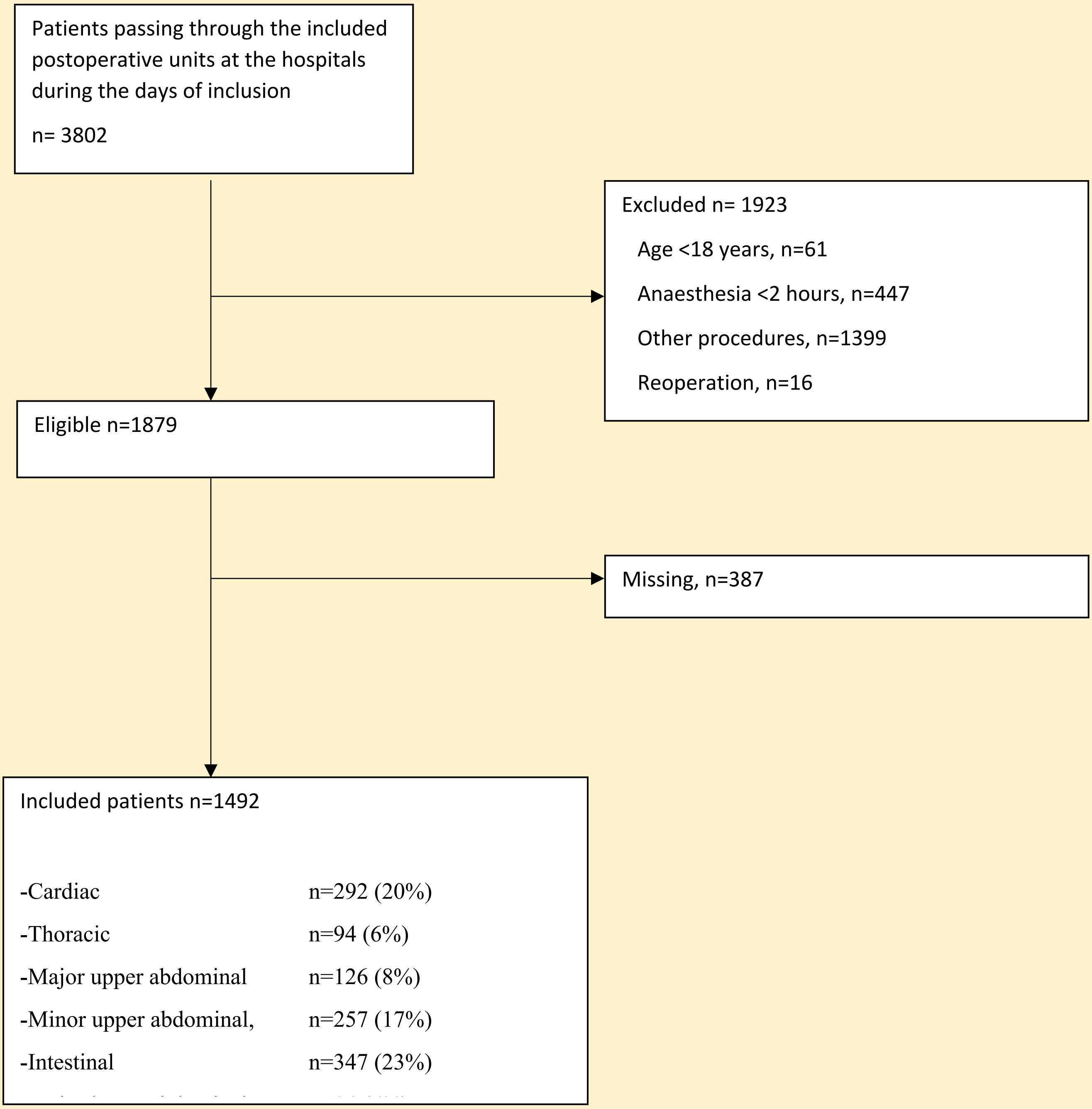

Figure 1. Flow chart of the patients included in the study.

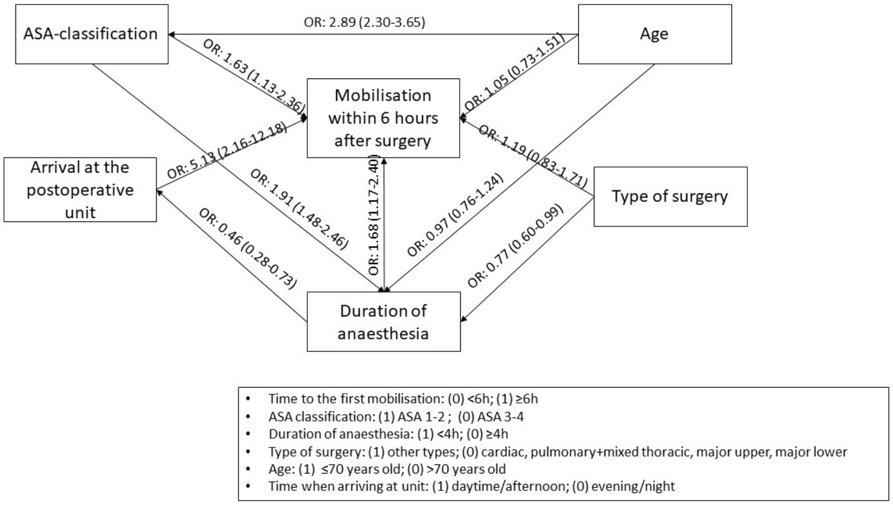

General structural equation model (GSEM) analysis displaying correlations between time to first mobilisation, categorised as within six hours and the factors; ASA-classification; age; arrival at the postoperative recovery unit; type of surgery; duration of surgery.

338x190mm (96 x 96 DPI)

Supplementary file

Table A

The 18 hospitals included in the study.

**University Hospitals**
-Karolinska University Hospital, Stockholm
-Linköpings University Hospital
-Sahlgrenska University Hospital, Gothenburg
-Skånes University Hospital, Lund, and Malmö
-University Hospital of Umeå
-Uppsala Akademiska Hospital
-Örebro University Hospital
**Regional County Hospitals**
-Centrallasarettet, Växjö
-Danderyd hospital, Stockholm
-Halmstad hospital
-Norra Älvsborgs Länssjukhus, Trollhättan
-Södersjukhuset, Stockholm
-Västerås hospital
-Östersund hospital
**Local County Hospitals**
-Alingsås hospital
-Höglandssjukhuset, Eksjö
-Visby hospital
-Örnsköldsviks hospital

For peer review only

Supplementary file

Supplementary file Table B: Odds ratios for mobilisation within three and 12 hours after abdominal or cardiothoracic surgery.

Variable	Value	Mobilisation within 3 hours			Mobilisation within 12 hours		
		n (%) of event	OR (95%CI)	P-value	n (%) of event	OR (95%CI)	P-value
**Category of surgery**	Reference: Cardiac	56 (19.2%)	1.00		204 (69.9%)	1.00	<0.0001***
	Thoracic	17 (18.5%)	0.96 (0.52-1.74)	0.88	60 (65.2%)	0.81 (0.49-1.33)	0.40
	Major upper abd	9 (7.4%)	0.34 (0.16-0.70)	0.0038	58 (47.5%)	0.39 (0.25-0.60)	<0.0001
	Minor upper abd	83 (34.3%)	2.20 (1.48-3.26)	<0.0001	188 (78.0%)	1.53 (1.03-2.27)	0.034
	Intestinal	67 (20.2%)	1.07 (0.72-1.59)	0.74	229 (69.4%)	0.98 (0.69-1.38)	0.90
	Major Lower Abd	15 (16.9%)	0.85 (0.46-1.60)	0.62	59 (66.3%)	0.85 (0.51-1.41)	0.52
	Minor Lower Abd	32 (12.1%)	0.58 (0.36-0.93)	0.024	199 (75.4%)	1.32 (0.91-1.92)	0.15
**Procedure**	Reference: Elective	236 (21.1%)	1.00		817 (73.0%)	1.00	<0.0001***
	Acute	21 (11.2%)	0.47 (0.29-0.76)	0.0019	100 (53.8%)	0.43 (0.31-0.59)	<0.0001
	Subacute	19 (16.2%)	0.73 (0.43-1.21)	0.22	75 (64.1%)	0.66 (0.44-0.98)	0.042
**Duration of anaesthesia**	Reference: 0-4 h	150 (22.6%)	0.68 (0.52-0.89)	0.0047	522 (78.7%)	0.44 (0.35-0.56)	<0.0001
	>4-	126 (16.6%)			469 (62.0%)		
**Arrival in postoperative care**	Reference: Day	155 (23.5%)	1.00		551 (83.5%)	1.00	<0.0001***
	Evening	122 (17.8%)	0.70 (0.54-0.92)	0.0099	390 (56.9%)	0.26 (0.20-0.34)	<0.0001
	Night	2 (2.3%)	0.08 (0.02-0.32)	0.0004	56 (65.9%)	0.38 (0.23-0.63)	0.0001
**Perioperative bleeding, ml**	Reference: <999 ml	179 (17.5%)	0.31 (0.14-0.73)		710 (69.6%)	0.47 (0.31-0.72)	0.0005
	≥1000 ml	6 (6.3%)		0.0070	50 (52.1%)		
**Epidural anaesthesia**	Reference: No	244 (21.3%)	0.48 (0.32-0.72)	0.0003	820 (71.9%)	0.57 (0.43-0.75)	<0.0001
	Yes	31 (11.5%)			159 (59.1%)		
**Spinal anaesthesia**	Reference: No	256 (20.1%)	0.62 (0.38-1.01)	0.056	885 (69.7%)	0.90 (0.63-1.30)	0.59
	Yes	20 (13.5%)			100 (67.6%)		
**Sex**	Reference: Male	152 (20.1%)	0.93 (0.71-1.21)	0.58	510 (67.5%)	1.24 (0.99-1.56)	0.063
	Female	127 (18.9%)			483 (72.1%)		
**Age, years**	Reference: 18-69	176 (20.6%)	0.83 (0.64-1.09)	0.18	625 (73.3%)	0.66 (0.53-0.83)	0.0004
	70-	103 (17.8%)			372 (64.5%)		
**Obesity**	Reference: BMI<30	196 (18.6%)	1.22 (0.91-1.64)	0.18	723 (68.9%)	1.20 (0.92-1.57)	0.18
	BMI>30	78 (21.9%)			258 (72.7%)		
**Functional status**	Reference: Independant	278 (19.6%)	0.26 (0.03-1.94)	0.19	991 (70.1%)	0.26 (0.09-0.71)	0.0088
	Dependant	1 (5.9%)			6 (37.5%)		
**Received preoperative information**	Reference: No	171 (22.8%)	0.60 (0.46-0.79)	0.0002	499 (66.8%)	1.30 (1.03-1.63)	0.026
	Yes	100 (15.1%)			480 (72.3%)		
**Type of hospital**	Reference university	150 (15.9%)	1.00		622 (66.0%)	1.00	<0.0001***
	Regional county	104 (25.6%)	1.82 (1.37-2.41)	<0.0001	299 (74.0%)	1.47 (1.13-1.90)	0.0040
	Local county	25 (29.8%)	2.24 (1.36-3.69)	0.0016	76 (90.5%)	4.89 (2.33-10.25)	<0.0001
**Type of ward**	Reference: IMU	7 (18.4%)	1.00		26 (68.4%)	1.00	0.0079***
	Postop/ICU	227 (28.0%)	1.72 (0.75-3.97)	0.20	614 (75.8%)	1.45 (0.72-2.92)	0.30
	Surgical ward	42 (8.2%)	0.40 (0.16-0.96)	0.039	348 (68.1%)	0.99 (0.49-2.00)	0.97

For peer review only

For peer review only - http://bmjopen.bmj.com/site/about/guidelines.xhtml

Abd: Abdominal, ICU: Intensive Care Unit, IMU: Intermediate Unit, OR: Odds Ratio, Postop: Postoperative Unit.

***) P-value for the entire effect/factor/variable

For peer review only

For peer review only - http://bmjopen.bmj.com/site/about/guidelines.xhtml

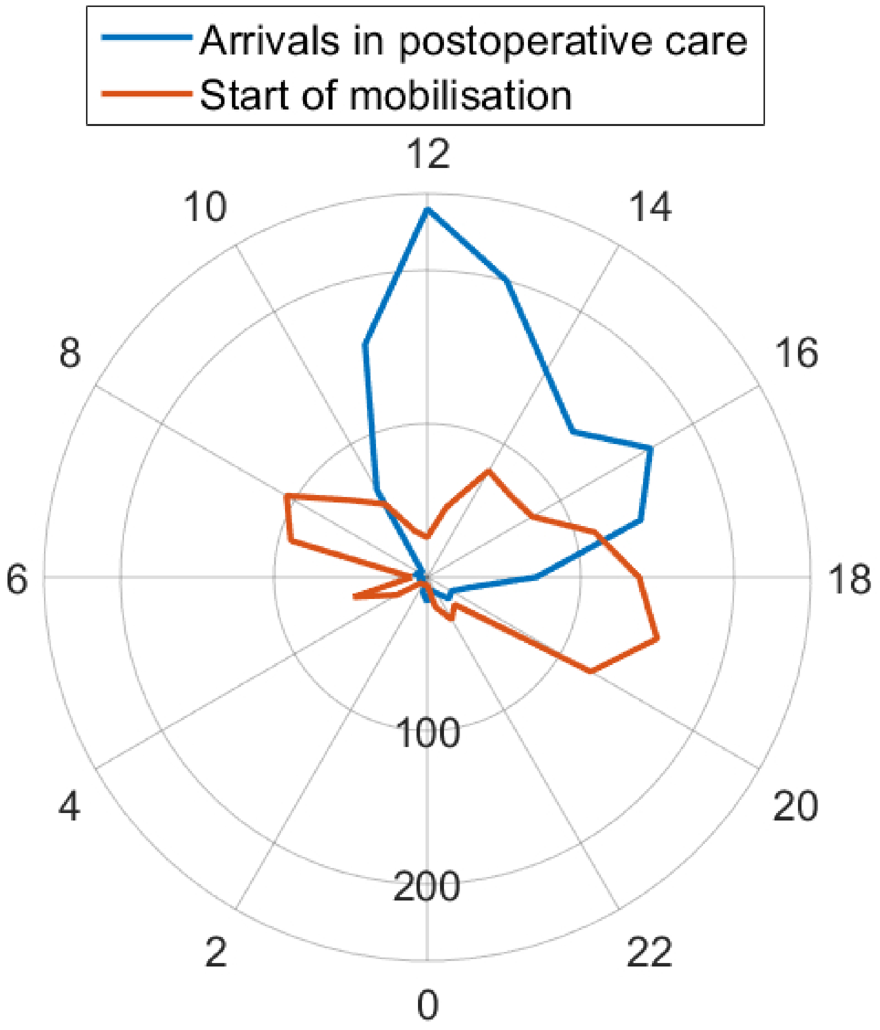

361x439mm (38 x 38 DPI)

STROBE Statement—Checklist of items that should be included in reports of ***cohort studies***

	Item No	Recommendation	Page No
**Title and abstract**	1	(*a*) Indicate the study's design with a commonly used term in the title or the abstract	Title
		(*b*) Provide in the abstract an informative and balanced summary of what was done and what was found	2
**Introduction**			
Background/rationale	2	Explain the scientific background and rationale for the investigation being reported	4
Objectives	3	State specific objectives, including any prespecified hypotheses	4
**Methods**			
Study design	4	Present key elements of study design early in the paper	5
Setting	5	Describe the setting, locations, and relevant dates, including periods of recruitment, exposure, follow-up, and data collection	5
Participants	6	(*a*) Give the eligibility criteria, and the sources and methods of selection of participants. Describe methods of follow-up<br>(*b*) For matched studies, give matching criteria and number of exposed and unexposed	5-6
Variables	7	Clearly define all outcomes, exposures, predictors, potential confounders, and effect modifiers. Give diagnostic criteria, if applicable	7
Data sources/ measurement	8*	For each variable of interest, give sources of data and details of methods of assessment (measurement). Describe comparability of assessment methods if there is more than one group	7
Bias	9	Describe any efforts to address potential sources of bias	5-7
Study size	10	Explain how the study size was arrived at	8<br>Fig1
Quantitative variables	11	Explain how quantitative variables were handled in the analyses. If applicable, describe which groupings were chosen and why	7-8
Statistical methods	12	(*a*) Describe all statistical methods, including those used to control for confounding<br>(*b*) Describe any methods used to examine subgroups and interactions<br>(*c*) Explain how missing data were addressed<br>(*d*) If applicable, explain how loss to follow-up was addressed<br>(*e*) Describe any sensitivity analyses	7-8
**Results**			
Participants	13*	(a) Report numbers of individuals at each stage of study—eg numbers potentially eligible, examined for eligibility, confirmed eligible, included in the study, completing follow-up, and analysed<br>(b) Give reasons for non-participation at each stage<br>(c) Consider use of a flow diagram	8<br>Fig1
Descriptive data	14*	(a) Give characteristics of study participants (eg demographic, clinical, social) and information on exposures and potential confounders<br>(b) Indicate number of participants with missing data for each variable of interest<br>(c) Summarise follow-up time (eg, average and total amount)	8<br>Table1
Outcome data	15*	Report numbers of outcome events or summary measures over time	9-10<br>Table2

For peer review only

For peer review only - http://bmjopen.bmj.com/site/about/guidelines.xhtml

Main results	16	(*a*) Give unadjusted estimates and, if applicable, confounder-adjusted estimates and their precision (eg, 95% confidence interval). Make clear which confounders were adjusted for and why they were included<br><br>(*b*) Report category boundaries when continuous variables were categorized<br>(*c*) If relevant, consider translating estimates of relative risk into absolute risk for a meaningful time period	9-10 Table2,3 Supplementary table Fig2,3
Other analyses	17	Report other analyses done—eg analyses of subgroups and interactions, and sensitivity analyses	9-10 Fig2, 3
**Discussion**			
Key results	18	Summarise key results with reference to study objectives	11
Limitations	19	Discuss limitations of the study, taking into account sources of potential bias or imprecision. Discuss both direction and magnitude of any potential bias	12-13
Interpretation	20	Give a cautious overall interpretation of results considering objectives, limitations, multiplicity of analyses, results from similar studies, and other relevant evidence	11-13
Generalisability	21	Discuss the generalisability (external validity) of the study results	13
**Other information**			
Funding	22	Give the source of funding and the role of the funders for the present study and, if applicable, for the original study on which the present article is based	14

*Give information separately for exposed and unexposed groups.

Note: An Explanation and Elaboration article discusses each checklist item and gives methodological background and published examples of transparent reporting. The STROBE checklist is best used in conjunction with this article (freely available on the Web sites of PLoS Medicine at http://www.plosmedicine.org/, Annals of Internal Medicine at http://www.annals.org/, and Epidemiology at http://www.epidem.com/). Information on the STROBE Initiative is available at http://www.strobe-statement.org.

For peer review only

For peer review only - http://bmjopen.bmj.com/site/about/guidelines.xhtml

BMJ Open

First mobilisation after abdominal and cardiothoracic surgery - When is it actually performed? A national, multicentre, cross-sectional study

Journal:	*BMJ Open*
Manuscript ID	bmjopen-2023-082239.R1
Article Type:	Original research
Date Submitted by the Author:	19-Jan-2024
Complete List of Authors:	Fagevik Olsén, Monika; University of Gothenburg, Neuroscience and Physiology; Sahlgrenska University Hospital, Physiotherapy<br>Sehlin, Maria; Umeå University, Department of Community Medicine and Rehabilitation<br>Westerdahl, Elisabeth; Orebro universitet<br>Schandl, Anna; Karolinska Institute<br>Block, Linda; Gothenburg University, Department of Anaesthesiology and Intensive Care<br>Nygren-Bonnier, Malin; Medical Unit Occupational Therapy and Physiotherapy, Department of Neurobiology, Care Sciences and Society<br>Svensson-Raskh, Anna; Karolinska Institutet, Department of Neurobiology, Care Sciences and Society, Division of Physiotherapy; Karolinska University Hospital, Department of Allied Health Professionals, Functional Area Occupational Therapy and Physiotherapy
**Primary Subject Heading**:	Surgery
Secondary Subject Heading:	Rehabilitation medicine
Keywords:	Cardiothoracic surgery < SURGERY, Physical Therapy Modalities, Colorectal surgery < SURGERY, Pancreatic surgery < SURGERY

SCHOLARONE™
Manuscripts

I, the Submitting Author has the right to grant and does grant on behalf of all authors of the Work (as defined in the below author licence), an exclusive licence and/or a non-exclusive licence for contributions from authors who are: i) UK Crown employees; ii) where BMJ has agreed a CC-BY licence shall apply, and/or iii) in accordance with the terms applicable for US Federal Government officers or employees acting as part of their official duties; on a worldwide, perpetual, irrevocable, royalty-free basis to BMJ Publishing Group Ltd ("BMJ") its licensees and where the relevant Journal is co-owned by BMJ to the co-owners of the Journal, to publish the Work in this journal and any other BMJ products and to exploit all rights, as set out in our licence.

The Submitting Author accepts and understands that any supply made under these terms is made by BMJ to the Submitting Author unless you are acting as an employee on behalf of your employer or a postgraduate student of an affiliated institution which is paying any applicable article publishing charge ("APC") for Open Access articles. Where the Submitting Author wishes to make the Work available on an Open Access basis (and intends to pay the relevant APC), the terms of reuse of such Open Access shall be governed by a Creative Commons licence – details of these licences and which Creative Commons licence will apply to this Work are set out in our licence referred to above.

Other than as permitted in any relevant BMJ Author's Self Archiving Policies, I confirm this Work has not been accepted for publication elsewhere, is not being considered for publication elsewhere and does not duplicate material already published. I confirm all authors consent to publication of this Work and authorise the granting of this licence.

For peer review only

ORIGINAL MANUSCRIPT

First mobilisation after abdominal and cardiothoracic surgery - When is it actually performed?

A national, multicentre, cross-sectional study

Monika Fagevik Olsén, PhD[1], Maria Sehlin, PhD[2], Elisabeth Westerdahl, PhD[3], Anna Schandl, PhD[4], Linda Block, PhD[5], Malin Nygren-Bonnier, PhD[6], Anna Svensson-Raskh, PhD[6]

[1] Department of Health and Rehabilitation/Physiotherapy, Institute of Neuroscience and Physiology and Department of Surgery, Institute of Clinical Sciences, Sahlgrenska Academy, University of Gothenburg, Gothenburg, Sweden. [2] Department of Community Medicine and Rehabilitation, Physiotherapy, Umeå University, Umeå, Sweden.[3]University Health Care Research Center and Department of Cardiothoracic and Vascular Surgery, Faculty of Medicine and Health, Örebro University, Örebro, Sweden. [4]Department of Anaesthesiology and Intensive Care, Södersjukhuset, Stockholm, Sweden/ Department of Clinical Science and Education, Karolinska Institutet, Södersjukhuset, Sweden. [5]Department of Anaesthesiology and Intensive Care, Institute of Clinical Sciences, Sahlgrenska Academy, Gothenburg University, Gothenburg, Sweden/ Department of Anaesthesiology and Intensive Care, Sahlgrenska University Hospital, Gothenburg, Sweden. [6]Department of Neurobiology, Care Sciences and Society, Division of Physiotherapy, Karolinska Institutet, Stockholm, Sweden/ Women´s Health and Allied Health Professionals Theme, Medical Unit Occupational Therapy and Physiotherapy, Karolinska University Hospital, Stockholm, Sweden

Short title: Mobilisation after surgery

Word-count main text: 2626

Keywords: Abdominal Surgery, Cardiothoracic Surgery, Mobilisation, Postoperative.

Corresponding author:

Professor Monika Fagevik Olsén

Dept. of Physical Therapy,

Sahlgrenska Academy,

Gothenburg University,

SE 413 45 Gothenburg, Sweden

e-mail: monika.fagevik-olsen@gu.se

Abstract

Objectives: Knowledge of clinical practice regarding mobilisation after surgery is lacking.

This study therefore aimed to reveal current mobilisation routines after abdominal and cardiothoracic surgery and to identify factors associated with mobilisation within six hours postoperatively.

Design: A prospective observational national multicentre study.

Setting: 18 different hospitals in Sweden.

Participants: 1492 adult patients undergoing abdominal and cardiothoracic surgery with duration of anaesthesia >2 hours.

Primary and secondary outcomes: Primary outcome was time to first postoperative mobilisation. Secondary outcomes were the type and duration of the first mobilisation. Data were analysed using multivariate logistic regression and general structural equation modelling, and data are presented as odds ratios (ORs) with 95% confidence intervals (CIs).

Results: Among the included patients, 52% were mobilised to at least sitting on the edge of the bed within six hours, 70% within 12 hours and 96% within 24 hours. Besides sitting on the edge of the bed, 76% stood up by the bed, and 22% were walking away from the bedside the first time they were mobilised. Patients undergoing major upper abdominal surgery required the longest time before mobilisation with an average time of 11 hours post-surgery. Factors associated with increased likelihood of mobilisation within six hours of surgery were daytime arrival at the postoperative recovery unit (OR 5.19, 95%; CI 2.16-12.18), anaesthesia <4 hours (OR 1.68, 95%; CI 1.17-2.41, and ASA classification 1-2, (OR 1.63, 95%; CI 1.13-2.36).

Conclusions: In total, 96% if the patients were mobilised within 24 hours after major surgery and 52% within six hours. Daytime arrival at the postoperative recovery unit, low ASA

For peer review only - http://bmjopen.bmj.com/site/about/guidelines.xhtml

classification, and shorter duration of anaesthesia were associated with a shorter time to mobilisation.

Trial registrations: FoU in VGR (Id:275357) and Clinical Trials (NCT04729634).

Strengths and limitations of this study:

- The prospective, multicentre design of this study, including patients from all parts of Sweden, and the relatively high participation rate counteract selection bias.
- The large study sample enabled sub-group analyses between the different types of surgery and exploration of related variables.
- Outcomes were determined by observations and relied on correct documentation by investigators.
- Patients were not recruited during weekends, and therefore the results may not completely reflect common practice.
- None of the mobilisations lead to any adverse events.

For peer review only - http://bmjopen.bmj.com/site/about/guidelines.xhtml

Background

Mobilisation after abdominal and cardiothoracic surgery is an important intervention for postoperative recovery and prevention of complications, even though its specific effects are largely unexplored (1,2). During decades of development of pre, peri and postoperative care, it has been found that other active interventions such as re-inflation of the lungs at the end of anaesthesia may reduce the risk of postoperative pulmonary complications (3-6). However, the specific effects of mobilisation are not yet known, nor how to facilitate it, nor when it should be initiated (7,8).

Undoubtedly, the change of position to sitting and/or standing has a multifactorial positive impact on cardiorespiratory function (8-10). Lung volume increases with a subsequent decrease of the atelectic area, improving the ventilation/perfusion ratio and facilitating the removal of secretions (11-13). The immediate respiratory effects of mobilisation have recently been evaluated in two randomised, controlled trials (14,15). In those, mobilisation within the first hours after surgery was compared to immobilised control groups. The results indicate that changing position from supine to upright has advantages for cardiorespiratory function and no major adverse events of the intervention were reported.

Most hospitals claim that “early” mobilisation is implemented in their standard practice of care, but the term “early” is not clearly defined (7), and the current state of practice remains unknown. Therefore, the present study aimed to estimate the time of first mobilisation, and to identify factors associated with a mobilisation within six hours in a large cohort of adult patients who had undergone acute, sub-acute, or elective abdominal or cardiothoracic surgery.

For peer review only - http://bmjopen.bmj.com/site/about/guidelines.xhtml

Materials and methods

Design and setting

This is a part of the prospective observational national multicentre cohort study entitled "Survey Of Mobilisation and Breathing exercise After cardio-Thoracic and Abdominal surgery (SOMBATA)". Eighteen hospitals representing all parts of Sweden participated, including seven university hospitals and eleven county or local hospitals, (see Supplementary Table 1). Data was collected between September 2021 and February 2022. The Swedish Ethical Review Authority approved the study (registration number 2020-03108) as well as all heads of the departments involved. Because of the observational study design of current practice, patient consent was waived.

Patient and public involvement

Patients or public were not involved in the design, or conduct, or reporting, or dissemination plans of our research.

Context of the study

In Sweden, most patients who have undergone abdominal and cardiothoracic surgery are treated and monitored at intensive care units (ICUs), specific postoperative units or intermediate units (IMA) (a lower level of care than ICU, but a higher level of care than surgical wards), for some hours until they are considered to have sufficient circulatory and respiratory stability to be transferred to surgical wards. The standard care includes mobilisation within the first day after surgery, assisted by nurses, assistant nurses, and physiotherapists. Some hospitals included in this study followed multimodal perioperative care pathways for some of the categories of patients.

For peer review only - http://bmjopen.bmj.com/site/about/guidelines.xhtml

Inclusion

Adult (≥ 18 years) patients who had undergone open, laparoscopic, or robot-assisted surgery within the thorax or abdomen, with a duration of anaesthesia exceeding two hours were eligible for inclusion. Patients who had undergone plastic, trauma, transplant, or orthopaedic surgery or reoperation were excluded. Data was captured on patients undergoing surgery from Monday to Thursday.

At each study site, one designated staff member (a physiotherapist or a nurse experienced in surgical practice) was responsible for inclusion screening of all patients and for controlling that all data was collected.

Data collection

Patient characteristics and preoperative variables were retrieved from the patients' records. Data on perioperative variables, such as surgical procedure, duration of anaesthesia (in hours and minutes), blood loss (ml), type of anaesthesia and functional status (partly/totally dependent) were obtained from the operation and postoperative surveillance charts. Type of surgery was categorised into: Cardiac (coronary artery bypass grafting, valve or aortic surgery via sternotomy); Thoracic (pulmonary surgery via thoracotomy or thoracoscopy); Major upper abdominal (pancreatic, hepatic, oesophageal and ventricular open procedures); Minor upper abdominal (open and laparoscopic procedures such as fundoplication and cholecystectomy); Intestinal (smaller intestine- and/or colorectal surgery); Major lower abdominal (extensive, open, gynaecological, urological, and other procedures with incision below the umbilicus); Minor lower abdominal (minor and laparoscopic gynaecological, urological, and other procedures with incision below the umbilicus).

The original research protocols were identifiable during the collection process to enable data completion from the patients´ records. When data was complete the protocols were de-identified before entering the results into a secure online database.

Outcomes

The primary outcome was time to first mobilisation from arrival at the postoperative unit. Mobilisation was defined as being able to at least sit on the edge of the bed. Secondary outcomes were type and duration of the first mobilisation, and number of staff assisting in mobilisation. Based upon the literature and clinical reasoning, the following data were considered potential factors influencing an early mobilisation (defined as within six hours of surgery), and were therefore collected; age, BMI, smoking, duration of anaesthesia, perioperative bleeding, co-morbidity (ASA classification), type of surgery, and arrival at the postoperative recovery unit (3,16-18).

The outcome data were registered, in parallel with the clinical work, in a case report form following the patient until 24 hours from admission to a postoperative unit. If no mobilisation was performed during the first 24 hours, the reason was noted in a bedside chart by the staff responsible for the trial at each study site. If any of the variables had been missed to be filled in a protocol these specific patient's results were excluded from the analysis.

Statistical Analysis

Continuous data were presented as means (standard deviation; SD) or median (interquartile range), while categorical data were illustrated with numbers and percentages [%]. Differences between groups (types of surgery) were analysed with ANOVA, Kruskal-Wallis test or Chi-square test.

The impact of baseline variables on the proportion of subjects and time to first mobilisation, time out of bed and type of mobilisation respectively, was analysed with univariable logistic regression. The significant univariable predictors were then entered stepwise into a multivariable logistic regression, using forward selection. Data were presented as odds ratios (ORs) with a 95% confidence interval (CI) and p-value.

A general structural equation model (GSEM) was applied to identify the associations between potential factors for being mobilised early and the time to first mobilisation, defined as within six hours from arrival at the postoperative unit (19). A univariate logistic model identified relevant factors: age, sex, BMI, perioperative bleeding, smoking, duration of anaesthesia, ASA classification, time of arrival at the postoperative recovery unit and type of surgery. Following this, a multivariate logistic regression was performed (including variables based on the statistical findings $p<0.05$), and to test for collinearity. The included variables were assessed stepwise and classified into the following categories: ASA classification (1-2 and 3-4) (11,17); age (≤70 and >70 years) (3,11,18), arrival at the postoperative recovery unit (daytime/afternoon and evening/night-time) (11); type of surgery (intestinal, minor upper and lower abdominal vs cardiac, thoracic, major upper and lower abdominal) (11,18); and duration of anaesthesia (<4 and ≥4 hours) (3,11,18). Finally, a GSEM was applied to analyse the structural association between the variables and mobilisation within six hours of arrival at the postoperative unit.

IBM SPSS version 29.0 and Stata Statistical Software: Release 17. College Station, TX, Stata Corp LLC were used for the analyses.

For peer review only - http://bmjopen.bmj.com/site/about/guidelines.xhtml

Results

Among 3802 patients who underwent abdominal or cardiothoracic surgery at the hospitals during the study periods, 1879 met the inclusion but not the exclusion criteria. Among them, 1492 patients (79%) were included in the study (Figure 1, Table 1).

Time to first mobilisation

Among the included patients, 279 (20%) were mobilised within three hours, 738 (52%) within six hours, 997 (70%) within 12 hours and 1396 (96%) within 24 hours. The mean time to the first mobilisation was 7 hrs 51 min (SD 6:27) (Table 2). Major upper abdominal surgery was associated with the longest time to mobilisation (mean 11 hrs 03 min, SD 4:08 hrs) whereas minor upper abdominal procedures were associated with the shortest time (mean 5 hrs 07 min, SD 5:38 hrs). Supplementary Figure 1 shows the times that patients arrived at the postoperative care units during the day and night and when the first mobilisation was started. Most patients arrived at the postoperative wards between 10:30 a.m. and 5:00 p.m. Most of the first mobilisations started around 8 a.m. and between 2 and 8 p.m.

Fifty-nine patients (4%) were not mobilised within the first 24 hours of surgery, and stated reasons (several reasons could be listed) were: circulatory instability (n=7), respiratory instability (n=15), sedation (n=12), intubation (n=6), pain (n=9), nausea (n=2), tiredness (n=7) or not considered a priority by the health care professionals (n=3). The reasons were similar across seven categories of surgery (data not shown).

Time out of bed and type of mobilisation

The mean duration of time out of bed was 27 min (SD 1 hour 16). Patients who had undergone cardiac surgery, intestinal surgery, and major lower abdominal surgery had the shortest time out of bed during their first mobilisation (Table 2).

Except that all patients were mobilised sat on the bed edge, 76% stood up by the bed, 15% sat in a chair and 22% walked around the first time they were out of bed. There were significant differences between the groups of surgical categories in all activities (p<0.001). When comparing the results between the seven types of surgery, the group that had cardiac surgery was the one that most often also were mobilised to standing up by the bed (89%), those that had lung surgery most often sat in a chair (82%), and those that underwent minor upper abdominal surgery were most often up and walked around (18%) (p<0.001). Commonly, two from the staff were assisting during the first mobilisation (Table 2). Most often a nurse (60%) or an assistant nurse (60%) assisted the patients during the mobilisation.

Factors associated with mobilisation.

The results of the odds ratio for mobilisation within six and 24 hours is presented in Table 3, and for three and 12 hours, see Supplementary Table 2. Minor upper abdominal surgery had the highest odds for mobilisation during the first six hours postoperatively (OR: 2.80, CI 1.96-3.98). Decreased odds were factors as acute surgery (OR:0.40, CI 0.29-0.55), arrival at the postoperative care unit during evenings (OR: 0.56 CI 0.45-0.70) and nights (OR 0.09, CI 0.05-0.18), duration of anaesthesia >4 hours (OR: 0.62, CI 0.50-0.76)) and bleeding >999 ml (OR: 0.48, CI 0.31-0.75)). The factors associated with increased odds of mobilisation even earlier (within three hours after surgery) were minor upper abdominal surgery (OR: 2.20, CI 1.48-3.26) and having surgery at a local county hospital (OR: 2.24, CI 1.36-3.69).

For peer review only - http://bmjopen.bmj.com/site/about/guidelines.xhtml

The strongest factors associated with increased odds of the first mobilisation within 24 hours were lower abdominal surgery (OR: 4.24, CI 0.55-32.86) (major surgery) and minor surgery (OR 3.09, CI 0.99-9.59), receiving preoperative information by the physiotherapist (OR: 4.03, CI 2.08-7.83) and surgery performed in local county hospitals (OR: 3.43, CI 0.47-25.31). Factors that decreased the odds were acute surgery and arriving to postoperative care during the night (OR: both 0.07).

According to the results of the GSEM (Figure 2), the following of the included factors were found to increase the probability of being mobilised within six hours of surgery (conditional on other factors): an ASA classification of 1 and 2 (OR: 1.63; 95% CI: 1.13 to 2.36), arriving at the postoperative recovery unit in the daytime/afternoon (OR: 5.13; 95% CI: 2.16 to 12.18) and a duration of anaesthesia of less than four hours (OR: 1.68; 95% CI: 1.17 to 2.40).

Discussion

This is the first prospective study describing mobilisation routines after cardiothoracic and abdominal surgery. In this national, multicentre, observational study, which included a total of 1492 patients, it was found that most patients (96%), were mobilised within the first 24 hours, whereas more than half of the cohort (52%) were mobilised within six hours. In addition to sitting on the edge of the bed, 76% stood up during the first mobilisation. Arriving at the postoperative recovery unit in the daytime, a duration of anaesthesia of less than four hours, and having few co-morbidities (ASA 1 or 2) were independently associated with being mobilised within six hours of surgery.

Even though almost all patients were mobilised within 24 hours there were some differences regarding the time to first mobilisation between the different types of surgery. Possible explanations are diverse methods of analgesia and different inflammatory responses after the surgical trauma (20) due to different proportions of a malign cause of surgery where cancer is

For peer review only - http://bmjopen.bmj.com/site/about/guidelines.xhtml

the most common cause of surgery for major upper abdominal surgery in contrary to cardiac surgery. The duration of first mobilisation also differed between the surgery types but not to the same extent as the time to mobilisation. The average time out of bed was 27 min but was about twice that after lung surgery. A shorter duration of anaesthesia during the pulmonary procedures via thoracotomy may be one explanatory factor compared to cardiac surgery via sternotomy or larger abdominal incision with longer duration of anaesthesia.

A more upright position appears to be essential to normalise functional residual capacity and respiratory function after anaesthesia and surgery (21-23). In addition, the upright position may also help to re-inflate the lung after atelectasis (12,13,24). An earlier and more intensive mobilization, i.e., to standing and walking, may therefore be preferable, especially for those at high risk of postoperative complications. In the current study, all mobilised patients sat on the edge of the bed, but a majority also stood up by the bed and 21 per cent even walked around. This is encouraging since the results from other studies show that compliance with Enhanced Recovery After Surgery (ERAS) recommendations is relatively poor with only 25-50% reaching the mobilisation targets after colorectal surgery (25,26). Nevertheless, there are few absolute contraindications for mobilisation, and as including more active approaches in clinical pathways has been proven to reduce complications and accelerate recovery (25,26) there may be room for improvement to fulfil the recommendations concerning mobilisation (7).

The findings of the present study indicate that arriving at the postoperative recovery unit in the daytime/afternoon is the strongest predictor of an earlier mobilisation (within six hours after surgery) as is a low ASA classification and a duration of anaesthesia of less than four hours. The reason why arrival during daytime is such a strong predictor is not known even if it is possible to speculate about variations in number of staff and differences in composition of professions during the day and night. Future studies are needed on this topic. However, what

For peer review only - http://bmjopen.bmj.com/site/about/guidelines.xhtml

is known is that early mobilization, including sitting in a chair, standing, and walking is not only feasible, but also leads to improved oxygenation as SpO_2 and PaO_2 in patients after pulmonary lobectomy (22) and different abdominal surgery (14,15,27). Thus, to facilitate early mobilisation after surgery, the focus might be to identify and target patients with comorbidities (ASA-classification ≥2) as it has been found that an ASA-class ≥2 is associated with an increased risk of postoperative pulmonary complications (28) In current trial, we did not investigate PPC, hence we do not know if patients with an early mobilisation developed fewer PPC compared to those with a late mobilisation, or whether mobilization itself constitutes a protective factor for PPC. Future trials are needed to explore that. Furthermore, to facilitate mobilisation it may be necessary to allocate all different healthcare professionals in the team at the postoperative recovery unit also to the evenings, when the patients with a prolonged surgery arrive (29,30).

In 1958, Powels (31) suggested that “early mobilisation” should occur on the first day after hernioplasty and “prompt mobilisation” should occur on the day of surgery. However, surgery as well as pre-, peri- and postoperative care have developed considerably since then. Despite the frequent use of “early” there is still no consensus about its definition[2, 7]. In the current trial, national practice after different surgeries was investigated. The results indicate that more than half of the patients were mobilised within six hours. It remains to be evaluated whether mobilisation within six hours after surgery may be used as a definition of early mobilisation in today’s practice. But to increase transparency and avoid misuse and misinterpretation of the term “early mobilisation” it is high time to discuss and come to a consensus about a definition.

For peer review only - http://bmjopen.bmj.com/site/about/guidelines.xhtml

Strengths and limitations

The prospective, multicentre design of this study, including patients from all parts of Sweden, and the relatively high participation rate counteract selection bias. To determine the standard practice of postoperative mobilisation, we aimed to include at least 700 patients, leaving approximately 100 patients in each surgical category on which we could perform sub-group analyses. The large study sample enabled the planned analyses of the different surgical categories and exploration of related variables. Still, approximately 20% of the included patients had no registered outcome data. Outcomes were determined by observations and relied on correct documentation by investigators. There may be several reasons for the missing data, such as lack of staff, problems reaching all staff with information about the study, and logistic challenges. Another limitation was that no patients were included during weekends, and therefore the results may not completely reflect common practice. Adverse events were not registered during the mobilisation. These would have given valuable information concerning the association between risk and when the mobilisation was performed. In addition, no score of frailty was included among the variables connected to mobilisation praxis but it is factor which could have had impact on the outcome. Future trials may also include a frailty index to further explore the subject.

Conclusion

Most of the patients in Sweden undergoing abdominal and cardiothoracic surgery that involved anaesthesia exceeding two hours were mobilised within the first 24 postoperative hours, with more than half mobilised within six hours, and the first mobilisation included standing in the majority of cases. Factors associated with earlier mobilisation were daytime arrival at the postoperative recovery unit, shorter duration of anaesthesia, and few co-

For peer review only - http://bmjopen.bmj.com/site/about/guidelines.xhtml

morbidities (low ASA classification). This overview of the national practice reveals unique baseline information to which other countries may be compared.

For peer review only - http://bmjopen.bmj.com/site/about/guidelines.xhtml

Funding statement: This study was financed by grants from the Swedish state under the agreement between the Swedish government and the county councils, the ALF-agreement (ALFGBG-965563), by The Local Research and Development Council, Gothenburg and Södra Bohuslän (VGFOUGSB-970106), and by Nyckelfonden, Örebro University Hospital Research Foundation (Grant number N/A).

Conflicts of interest: The authors declare no conflicts of interest.

Author contributions:

MFO had full access to all the data in the study and was responsible for the integrity of the data and the accuracy of the data analysis. Concept and design: MFO, MS, EW, AS, LB, MNB, ASR. Acquisition and analysis: MFO, ASR Interpretation of results: MFO, MS, EW, AS, LB, MNB, ASR Critical revision of the manuscript: MFO, MS, EW, AS, LB, MNB, ASR Statistical analysis: MFO, ASR and statistical consultants Guarantor: Gothenburg University

Data sharing statement*:* Data, Materials and Analytical Methods will be shared on request.

Ethics approval: The Swedish Ethical Review Authority approved the study (registration number 2020-03108).

For peer review only - http://bmjopen.bmj.com/site/about/guidelines.xhtml

Figure legends:

Figure 1. Flow chart of the patients included in the study

Figure 2. General structural equation model (GSEM) analysis displaying correlations between time to first mobilisation, categorised as within six hours and the factors; ASA-classification; age; arrival at the postoperative recovery unit; type of surgery; duration of surgery.

Supplementary Figure 1. Supplementary. Number of participants arriving in postoperative care and number of participants starting mobilisation during the night and day.

For peer review only - http://bmjopen.bmj.com/site/about/guidelines.xhtml

References

1. Ljungqvist O, Scott M, Fearon KC. Enhanced Recovery After Surgery: A Review. JAMA Surgery 2017;152: 292-8
2. Tazreean R, Nelson G, Twomey R. Early mobilization in enhanced recovery after surgery pathways: current evidence and recent advancements. J Comp Eff Res 2022;11: 121-9
3. Gallart L, Canet J. Post-operative pulmonary complications: Understanding definitions and risk assessment. Best practice & research Clinical anaesthesiology 2015;29: 315-30
4. Westerdahl E, Lindmark B, Eriksson T, et al. Deep-breathing exercises reduce atelectasis and improve pulmonary function after coronary artery bypass surgery. Chest 2005;128: 3482-8
5. Yang D, Grant MC, Stone A, et al. A Meta-analysis of Intraoperative Ventilation Strategies to Prevent Pulmonary Complications: Is Low Tidal Volume Alone Sufficient to Protect Healthy Lungs? Ann Surg 2016;263: 881-7
6. Odor PM, Bampoe S, Gilhooly D, et al. Perioperative interventions for prevention of postoperative pulmonary complications: systematic review and meta-analysis. BMJ 2020;368: m540
7. Castelino T, Fiore JF, Jr., Niculiseanu P, et al. The effect of early mobilization protocols on postoperative outcomes following abdominal and thoracic surgery: A systematic review. Surgery 2016;159: 991-1003
8. Ramos Dos Santos PM, Aquaroni Ricci N, et al. Effects of early mobilisation in patients after cardiac surgery: a systematic review. Physiotherapy 2017;103: 1-12.
9. Hsu HO, Hickey RF. Effect of posture on functional residual capacity postoperatively. Anesthesiology 1976;44: 520-1
10. Katz S, Arish N, Rokach A, et al. The effect of body position on pulmonary function: a systematic review. BMC Pulm Med 2018;18: 159
11. Miskovic A, Lumb AB. Postoperative pulmonary complications. Br J Anaesth 2017;118: 317-34
12. Moradian ST, Najafloo M, Mahmoudi H, Ghiasi MS. Early mobilization reduces the atelectasis and pleural effusion in patients undergoing coronary artery bypass graft surgery: A randomized clinical trial. J Vasc Nurs 2017;35: 141-5
13. Hanada M, Kanetaka K, Hidaka S, et al. Effect of early mobilization on postoperative pulmonary complications in patients undergoing video-assisted thoracoscopic surgery on the esophagus. Esophagus 2018;15: 69-74
14. Svensson-Raskh A, Schandl AR, Ståhle A, et al. Mobilization Started Within 2 Hours After Abdominal Surgery Improves Peripheral and Arterial Oxygenation: A Single-Center Randomized Controlled Trial. Phys Ther 2021;101
15. Fagevik Olsén M, Becovic S, Dean E. Short-term effects of mobilization on oxygenation in patients after open surgery for pancreatic cancer: a randomized controlled trial. BMC Surgery 2021;21: 185
16. Fernandez-Bustamante A, Frendl G, Sprung J, et al. Postoperative Pulmonary Complications, Early Mortality, and Hospital Stay Following Noncardiothoracic Surgery: A Multicenter Study by the Perioperative Research Network Investigators. JAMA Surg 2017;152: 157-66
17. Smetana GW, Lawrence VA, Cornell JE. Preoperative pulmonary risk stratification for noncardiothoracic surgery: systematic review for the American College of Physicians. Ann Inter Med 2006;144: 581-95
18. Neto AS, da Costa LGV, Hemmes SNT, et al. The LAS VEGAS risk score for prediction of postoperative pulmonary complications: An observational study. Eur J Anaesthesiol 2018;35: 691-701
19. Armstrong RA. Recommendations for analysis of repeated-measures designs: testing and correcting for sphericity and use of manova and mixed model analysis. Ophthalmic and Physiological Optics. 2017;37: 585-93
20. Giannoudis PV, Dinopoulos H, Chalidis B, Hall GM. Surgical stress response. 2006;37 Suppl 5: S3-9

For peer review only - http://bmjopen.bmj.com/site/about/guidelines.xhtml

21. Nielsen KG, Holte K, Kehlet H. Effects of posture on postoperative pulmonary function. Acta Anaesthesiol Scand 2003;47: 1270-5
22. Kaneda H, Saito Y, Okamoto M, et al. Early postoperative mobilization with walking at 4 hours after lobectomy in lung cancer patients. Gen Thorac Cardiovasc Surg 2007;55: 493-8
23. Mynster T, Jensen LM, Jensen FG, et al. The effect of posture on late postoperative oxygenation. Anaesthesia 1996;51: 225-7
24. Lindberg P, Gunnarsson L, Tokics L, et al. Atelectasis and lung function in the postoperative period. Acta Anaesthesiol Scand 1992;36: 546-53
25. Grass F, Pache B, Martin D, et al. Feasibility of early postoperative mobilisation after colorectal surgery: A retrospective cohort study. Int J Surgery 2018;56: 161-6
26. Gustafsson UO, Hausel J, Thorell A, et al. Adherence to the enhanced recovery after surgery protocol and outcomes after colorectal cancer surgery. Arch Surg 2011;146: 571-7
27. Thörn RW, Stepniewski J, Hjelmqvist H, et al. Supervised Immediate Postoperative Mobilization After Elective Colorectal Surgery: A Feasibility Study. World J Surg 2022;46: 34-42
28. Sameed M, Choi H, Auron M, Mireles-Cabodevila E. Preoperative Pulmonary Risk Assessment. Respir Care. 2021;66(7):1150-1166
29. Garzon-Serrano J, Ryan C, Waak K, et al. Early mobilization in critically ill patients: patients' mobilization level depends on health care provider's profession. PM & R 2011;3: 307-13
30. Fletcher N, Engelman DT. Postoperative care: who should look after patients following surgery? Anaesthesia.2020;75: e5-e9
31. Powers JH. Early ambulation: its influence on postoperative complications and return to work following hernioplasty in a rural population. Ann N Y Acad Sci 1958;73: 524-36

For peer review only - http://bmjopen.bmj.com/site/about/guidelines.xhtml

Table 1. Demographic data of the included patients. Data presented as mean (SD) or n [%].

	All, n=1492	Cardiac, n=292	Thoracic n=94	Major upper abd, n=126	Minor upper abd, n=257	Intestinal n=347	Major lower abd n=94	Minor lower abd n=282
Preoperative factors								
Age, years	62 (16)	64 (14)	60 (18)	67 (13)	56 (18)	67 (16)	62 (14)	60 (15)
Male sex, n	784 (49.1%)	227 (77.7%)	49 (52.1%)	81 (64.3%)	96 (37.4%)	175 (50.4%)	33 (35.1%)	123 (43.6%)
Body Mass Index, kg/m^2	27.7 (5.5)	27.0 (4.9)	25.7 (4.5)	27.0 (4.8)	29.1 (6.4)	26.3 (5.8)	26.4 (5.1)	27.0 (5.3)
Functional status- partly/totally dependent, n	18 (1.1%)	2 (0.7%)	0	1 (0.8%)	5 (1.9%)	9 (2.6%)	0	1 (0.4%)
ASA, score								
1	171 (11.8%)	0	6 (6.4%)	8 (6.8%)	42 (17.1%)	30 (8.8%)	14 (14.9%)	71 (25.5%)
2	575 (39.5%)	12 (4.2%)	37 (39.4%)	53 (44.9%)	119 (48.4%)	156 (46.0%)	64 (68.1%)	134 (48.2%)
3	611 (42.0%)	212 (74.4%)	48 (51.1%)	56 (47.5%)	73 (28.4%)	136 (40.1%)	15 (16.0%)	71 (25.5%)
4	96 (6.6%)	60 (21.1%)	3 (3.2%)	1 (0.8%)	12 (4.7%)	17 (5.0%)	1 (1.1%)	2 (0.7%)
5	1 (0.1%)	1 (0.4%)	0	0	0	0	0	0
Missing	38	7	0	8	11	8	0	4
Perioperative factors								
Duration of anaesthesia, h	4.60 (2.17)	4.80 (1.46)	3.366 (1.12)	7.22 (3.12)	3.43 (1.35)	5.26 (2.45)	4.72 (1.81)	3.77 (1.41)

For peer review only - http://bmjopen.bmj.com/site/about/guidelines.xhtml

Duration of surgery, h	3.27 (2.16)	3.41 (1.95)	2.03 (1.07)	5.53 (2.98)	2.33 (1.20)	3.90 (2.29)	3.34 (1.73)	2.59 (1.74)
Open surgery, n	826 (55.6%)	282 (96.9%)	31 (33.0%)	102 (81.6%)	68 (26.7%)	195 (53.5%)	60 (63.8%)	88 (31.3%)

ASA: American Society of Anaesthesiologists Classification

Table 2. Time to first mobilisation, duration, and type of mobilisation, presented as mean (SD), median (Q1:Q3) or n [per cent, %].

	All, n=1492	Cardiac, n=292	Thoracic n=94	Major upper abd n=126	Minor upper abd, n=257	Intestinal n=347	Major lower abd n=94	Minor lower abd n=282
Time to first mobilisation, h*	7.91 (5.93) 5.50 (3.30:13.02)	8.20 (5.47) 6.67 (4.00:12.50)	8.39 (6.61) 4.92 (3.31:15.06)	11.05 (5.80) 11.80 (4.84:16.29)	5.95 (5.63) 3.75 (2.22:7.41)	7.48 (8.02) 5.25 (3.25:13.72)	8.75 (6.21) 6.10 (3.31:15.70)	7.63 (5.68) 2.26 (3.92:8.71)
Time to first mobilisation, categorised in intervals, hrs*								
<3	279 (20.4%)	56 (20.1%)	15 (16.9%)	11 (9.3%)	83 (36.6%)	67 (21.6%)	15 (17.0%)	32 (12.4%)
3-<6	458 (33.4%)	64 (22.9%)	39 (44.0%)	25 (21.2%)	76 (33.5%)	111 (35.8%)	28 (31.8%)	115 (44.4%)
6-<9	186 (13.6%)	55 (19.7%)	4 (4.5%)	9 (7.6%)	18 (7.9%)	36 (11.6%)	14 (15.9%)	50 (19.3%)
9-<12	74 (5.4%)	29 (10.4%)	5 (5.6%)	12 (10.2%)	9 (4.0%)	15 (4.8%)	2 (2.2%)	2 (0.8%)
12-<15	103 (7.5%)	30 (10.8%)	8 (9.0%)	20 (16.9%)	11 (4.8%)	18 (5.8%)	3 (3.4%)	13 (5.0%)
15-<18	160 (11.7%)	30 (10.8%)	10 (11.2%)	28 (23.9%)	15 (6.6%)	35 (11.3%)	18 (20.5%)	24 (9.3%)
18-<21	87 (6.4%)	15 (5.4%)	3 (3.4%)	11 (9.3%)	12 (5.3%)	22 (7.1%)	7 (8.0%)	17 (6.6%)
21-24	23 (1.8%)	0	5 (5.6%)	2 (1.8%)	3 (1.3%)	6 (1.8%)	1	6 (2.3%)
No time reg.	26	0	2	2	6	7	3	6
Not mobilised	62	13	2	5	15	22	1	4
Missing	34	0	1	1	9	8	2	13
Mobilised within 24 hrs, n	1430 (95.8%)	279 (95.9%)	91 (97.8%)	120 (96.0%)	233 (94.0%)	317 (94.3%)	91 (98.9%)	265 (98.5%)
Duration of mobilisation, h*	0.45 (1.27) 0.17 (0.17:0.33)	0.34 (0.68) 1.67 (0.08:0.25)	0.96 (2.55) 0.50 (0.17:1.00)	0.49 (0.69) 0.25 (0.17:0.50)	0.58 (1.82) 0.25 (0.17:0.48)	0.31 (0.41) 0.67 (0.67:0.33)	0.27 (0.29) 0.67 (0.12:0.30)	0.47 (1.56) 0.17 (0.08-0.33)

Sitting on the edge of the bed	1395 [100]	279 [100]	91 [100]	119 [100]	234 [100]	317 [100]	91 [100]	265 [100]
Standing by the bed*	1059 [75.9]	248 [88.9]	75 [82.4]	85 [71.4]	182 [70.8]	197 [62.1]	73 [80.2]	199 [75.4]
Sitting in a chair*	203 [14.6]	42 [15.1]	47 [51.6]	31 [26.1]	23 [9.8]	31 [8.9]	10 [11.0]	19 [7.2]
Walking*	294 [21.1]	1 [0.4]	17 [18.7]	8 [6.7]	112 [47.9]	42 [13.2]	19 [20.9]	95 [36.1]
Distance walked, m*	8.3 (5.8)	7.5 (10.6)	45.9 (46.8)	33.3 (32.7)	23.0 (26.3)	32.3 (32.0)	56.7 (82.2)	40.5 (53.6)
Number of staff assisting, n*								
0	31 (2.4%)	0	0	0	4 (1.9%)	1 (0.3%)	4 (5.1%)	22 (9.6%)
1	406 (31.6%)	4 (1.5%)	26 (30.2%)	14 (12.3%)	135 (62.8%)	72 (24.8%)	30 (38.5%)	125 (54.3%)
2	732 (57.0%)	223 (82.2%)	57 (66.3%)	74 (64.9%)	72 (33.5%)	192 (66.2%)	36 (46.2%)	78 (33.9%)
3	103 (8.0%)	40 (14.8%)	3 (3.5%)	22 (19.3%)	4 (1.9%)	24 (8.3%)	6 (7.6%)	4 (1.7%)
4	12 (0.9%)	4 (1.5%)	0 (86)	4 (3.5%)	0	1 (0.3%)	2 (2.6%)	1 (0.4%)

*Difference between the seven types of surgery=p<0.001,

Abd: Abdominal, Reg: Registered, Q: Quartile

Table 3. Odds ratios for mobilisation within six and 24 hours after abdominal or cardiothoracic surgery.

Variable	Value	Mobilisation within 6 hours			Mobilisation within 24 hours		
		n (%) of event	OR (95%CI)	P-value	n (%) of event	OR (95%CI)	P-value
**Category of surgery**	Reference: Cardiac	120 (41.1%)	1.00	<0.0001***	279 (95.5%)	1.00	0.12***
	Thoracic	55 (59.8%)	2.13 (1.32-3.43)	0.0019	91 (97.8%)	2.12 (0.47-9.57)	0.33
	Major upper abd	34 (27.9%)	0.55 (0.35-0.88)	0.012	118 (95.2%)	0.92 (0.34-2.47)	0.86
	Minor upper abd	160 (66.1%)	2.80 (1.96-3.98)	<0.0001	234 (94.4%)	0.78 (0.36-1.69)	0.53
	Intestinal	179 (54.1%)	1.69 (1.23-2.32)	0.0012	317 (94.3%)	0.78 (0.38-1.60)	0.50
	Major Lower Abd	43 (48.3%)	1.34 (0.83-2.16)	0.23	91 (98.9%)	4.24 (0.55-32.86)	0.17
	Minor Lower Abd	147 (55.7%)	1.80 (1.29-2.52)	0.0006	265 (98.5%)	3.09 (0.99-9.59)	0.051
**Procedure**	Reference: Elective	620 (55.4%)	1.00	<0.0001***	1119 (98.5%)	1.00	<0.0001***
	Acute	62 (33.0%)	0.40 (0.29-0.55)	<0.0001	155 (81.6%)	0.07 (0.04-0.12)	<0.0001
	Subacute¤	52 (44.4%)	0.64 (0.44-0.94)	0.024	112 (94.1%)	0.24 (0.10-0.60)	0.0021
**Duration of anaesthesia**	Reference: 0-4 h	385 (58.0%)	0.62 (0.50-0.76)	<0.0001	651 (96.7%)	0.71 (0.41-1.22)	0.22
	>4-	349 (46.0%)			736 (95.5%)		
**Arrival in postoperative care**	Reference: Day	404 (61.2%)	1.00	<0.0001***	652 (97.8%)	1.00	<0.0001***
	Evening	323 (47.1%)	0.56 (0.45-0.70)	<0.0001	676 (96.8%)	0.71 (0.36-1.37)	0.31
	Night	11 (12.8%)	0.09 (0.05-0.18)	<0.0001	67 (75.3%)	0.07 (0.03-0.14)	<0.0001
**Perioperative bleeding, ml**	Reference: <999 ml	509 (49.9%)	0.48 (0.31-0.75)		999 (96.3%)	0.58 (0.24-1.40)	0.22
	≥1000 ml	31 (32.3%)		0.0012	91 (93.8%)		
**Epidural anaesthesia**	Reference: No	606 (53.0%)	0.68 (0.52-0.89)	0.0051	1115 (96.0%)	0.82 (0.44-1.54)	0.54
	Yes	117 (43.5%)			259 (95.2%)		
**Spinal anaesthesia**	Reference: No	653 (51.4%)	0.97 (0.69-1.37)	0.87	1233 (95.7%)	2.24 (0.69-7.25)	0.18
	Yes	75 (50.7%)			148 (98.0%)		
**Sex**	Reference: Male	373 (49.3%)	1.20 (0.97-1.47)	0.093	732 (95.6%)	1.22 (0.72-2.07)	0.45
	Female	361 (53.8%)			658 (96.3%)		
**Age, years**	Reference: 18-69	461 (54.0%)	0.78 (0.63-0.96)	0.021	842 (97.1%)	0.48 (0.28-0.82)	0.0068

Variable	Value	Mobilisation within 6 hours			Mobilisation within 24 hours		
		n (%) of event	OR (95%CI)	P-value	n (%) of event	OR (95%CI)	P-value
	70-	277 (47.8%)			553 (94.2%)		
**Obesity**	Reference: BMI<30	534 (50.8%)	1.17 (0.92-1.49)	0.20	1026 (96.2%)	1.14 (0.59-2.20)	0.69
	BMI>30	195 (54.8%)			351 (96.7%)		
**Functional status**	Reference: Independant	735 (51.9%)	1.00		1382 (96.2%)	0.10 (0.03-0.30)	<0.0001
	Dependant	3 (17.6%)	0.20 (0.06-0.69)	0.011	13 (72.2%)		
**Received preoperative information**	Reference: No	392 (52.3%)	0.90 (0.73-1.11)	0.32	715 (93.7%)	4.03 (2.08-7.83)	<0.0001
	Yes	330 (49.7%)			661 (98.4%)		
**Type of hospital**	Reference university	448 (47.6%)	1.00	<0.0001***	919 (96.0%)	1.00	0.33***
	Regional county	232 (57.1%)	1.47 (1.16-1.86)	0.0013	393 (95.2%)	0.81 (0.47-1.41)	0.46
	Local county	58 (69.0%)	2.46 (1.52-3.97)	0.0002	83 (98.8%)	3.43 (0.47-25.31)	0.23
**Type of ward**	Reference: IMU	23 (60.5%)	1.00	0.0005***			
	Postop/ICU	468 (57.8%)	0.89 (0.46-1.74)	0.74			
	Surgical ward	240 (47.0%)	0.58 (0.29-1.13)	0.11			

Abd: Abdominal, ICU: Intensive Care Unit, IMU: Intermediate Unit, OR: Odds Ratio, Postop: Postoperative Unit. ¤ Acute surgery was defined as surgery within 24 hours after decision of surgery and subacute surgery within a week after the decision.

***) P-value for the entire effect/factor/variable

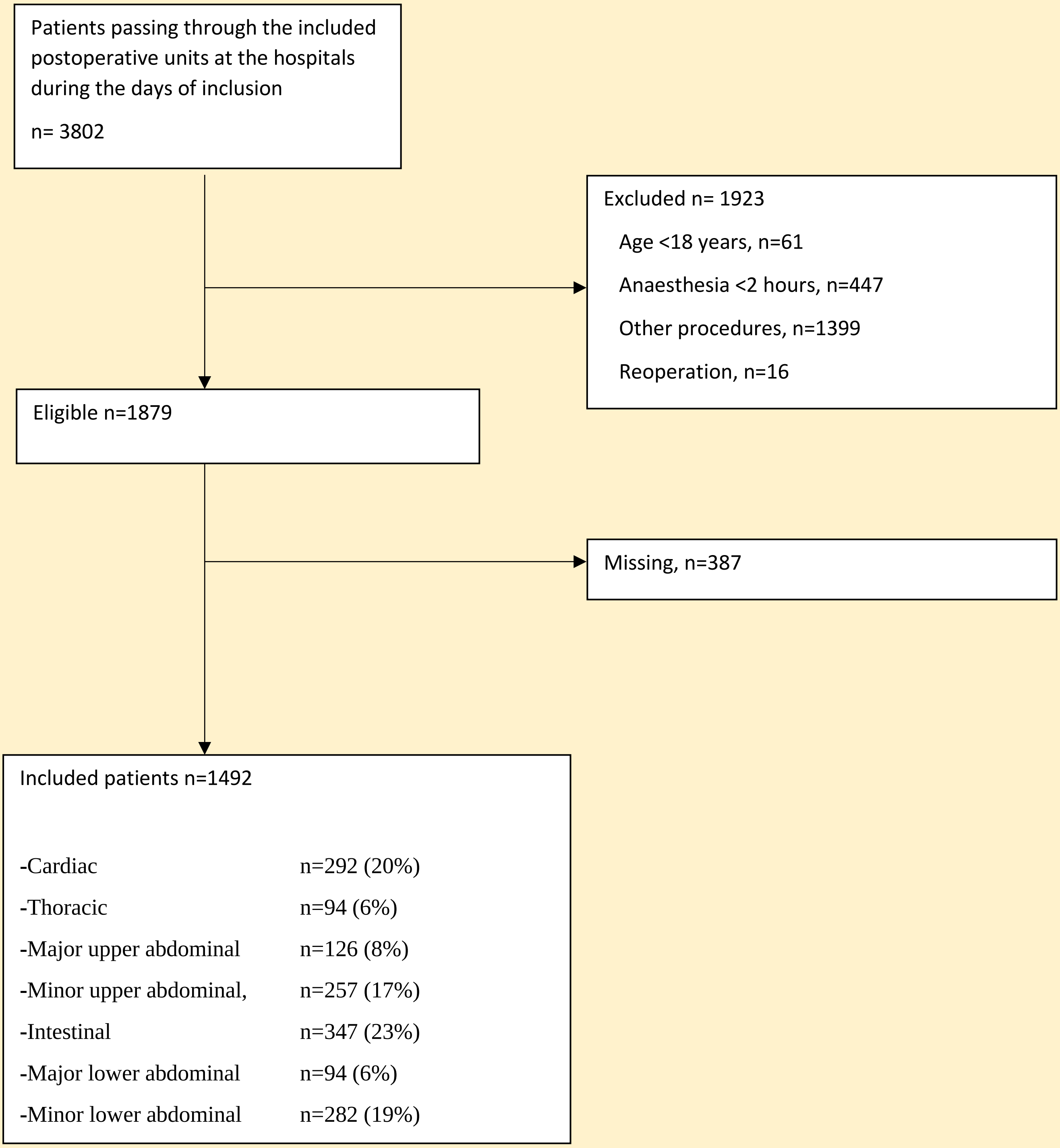

Figure 1. Flow chart of the patients included in the study.

For peer review only - http://bmjopen.bmj.com/site/about/guidelines.xhtml

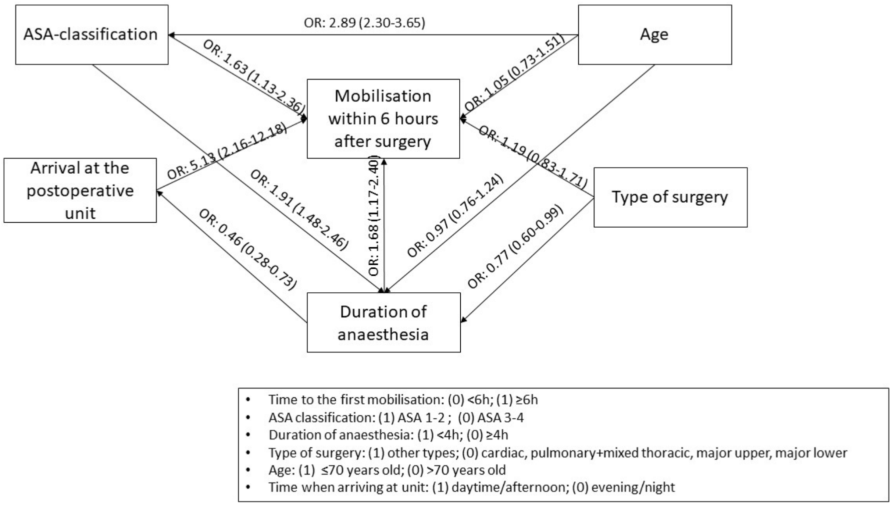

General structural equation model (GSEM) analysis displaying correlations between time to first mobilisation, categorised as within six hours and the factors; ASA-classification; age; arrival at the postoperative recovery unit; type of surgery; duration of surgery.

338x190mm (96 x 96 DPI)

Supplementary Table 1

The 18 hospitals included in the study.

University Hospitals
-Karolinska University Hospital, Stockholm
-Linköpings University Hospital
-Sahlgrenska University Hospital, Gothenburg
-Skånes University Hospital, Lund, and Malmö
-University Hospital of Umeå
-Uppsala Akademiska Hospital
-Örebro University Hospital

Regional County Hospitals
-Centrallasarettet, Växjö
-Danderyd hospital, Stockholm
-Halmstad hospital
-Norra Älvsborgs Länssjukhus, Trollhättan
-Södersjukhuset, Stockholm
-Västerås hospital
-Östersund hospital

Local County Hospitals
-Alingsås hospital
-Höglandssjukhuset, Eksjö
-Visby hospital
-Örnsköldsviks hospital

For peer review only

Table 4: Odds ratios for mobilisation within three and 12 hours after abdominal or cardiothoracic surgery.

Variable	Value	Mobilisation within 3 hours			Mobilisation within 12 hours		
		n (%) of event	OR (95%CI)	P-value	n (%) of event	OR (95%CI)	P-value
**Category of surgery**	Reference: Cardiac	56 (19.2%)	1.00		204 (69.9%)	1.00	<0.0001***
	Thoracic	17 (18.5%)	0.96 (0.52-1.74)	0.88	60 (65.2%)	0.81 (0.49-1.33)	0.40
	Major upper abd	9 (7.4%)	0.34 (0.16-0.70)	0.0038	58 (47.5%)	0.39 (0.25-0.60)	<0.0001
	Minor upper abd	83 (34.3%)	2.20 (1.48-3.26)	<0.0001	188 (78.0%)	1.53 (1.03-2.27)	0.034
	Intestinal	67 (20.2%)	1.07 (0.72-1.59)	0.74	229 (69.4%)	0.98 (0.69-1.38)	0.90
	Major Lower Abd	15 (16.9%)	0.85 (0.46-1.60)	0.62	59 (66.3%)	0.85 (0.51-1.41)	0.52
	Minor Lower Abd	32 (12.1%)	0.58 (0.36-0.93)	0.024	199 (75.4%)	1.32 (0.91-1.92)	0.15
**Procedure**	Reference: Elective	236 (21.1%)	1.00		817 (73.0%)	1.00	<0.0001***
	Acute ¤	21 (11.2%)	0.47 (0.29-0.76)	0.0019	100 (53.8%)	0.43 (0.31-0.59)	<0.0001
	Subacute ¤	19 (16.2%)	0.73 (0.43-1.21)	0.22	75 (64.1%)	0.66 (0.44-0.98)	0.042
**Duration of anaesthesia**	Reference: 0-4 h	150 (22.6%)	0.68 (0.52-0.89)	0.0047	522 (78.7%)	0.44 (0.35-0.56)	<0.0001
	>4-	126 (16.6%)			469 (62.0%)		
**Arrival in postoperative care**	Reference: Day	155 (23.5%)	1.00		551 (83.5%)	1.00	<0.0001***
	Evening	122 (17.8%)	0.70 (0.54-0.92)	0.0099	390 (56.9%)	0.26 (0.20-0.34)	<0.0001
	Night	2 (2.3%)	0.08 (0.02-0.32)	0.0004	56 (65.9%)	0.38 (0.23-0.63)	0.0001
**Perioperative bleeding, ml**	Reference: <999 ml	179 (17.5%)	0.31 (0.14-0.73)		710 (69.6%)	0.47 (0.31-0.72)	0.0005
	≥1000 ml	6 (6.3%)		0.0070	50 (52.1%)		
**Epidural anaesthesia**	Reference: No	244 (21.3%)	0.48 (0.32-0.72)	0.0003	820 (71.9%)	0.57 (0.43-0.75)	<0.0001
	Yes	31 (11.5%)			159 (59.1%)		
**Spinal anaesthesia**	Reference: No	256 (20.1%)	0.62 (0.38-1.01)	0.056	885 (69.7%)	0.90 (0.63-1.30)	0.59
	Yes	20 (13.5%)			100 (67.6%)		
**Sex**	Reference: Male	152 (20.1%)	0.93 (0.71-1.21)	0.58	510 (67.5%)	1.24 (0.99-1.56)	0.063
	Female	127 (18.9%)			483 (72.1%)		
**Age, years**	Reference: 18-69	176 (20.6%)	0.83 (0.64-1.09)	0.18	625 (73.3%)	0.66 (0.53-0.83)	0.0004
	70-	103 (17.8%)			372 (64.5%)		
**Obesity**	Reference: BMI<30	196 (18.6%)	1.22 (0.91-1.64)	0.18	723 (68.9%)	1.20 (0.92-1.57)	0.18
	BMI>30	78 (21.9%)			258 (72.7%)		
**Functional status**	Reference: Independant	278 (19.6%)	0.26 (0.03-1.94)	0.19	991 (70.1%)	0.26 (0.09-0.71)	0.0088
	Dependant	1 (5.9%)			6 (37.5%)		
**Received preoperative information**	Reference: No	171 (22.8%)	0.60 (0.46-0.79)	0.0002	499 (66.8%)	1.30 (1.03-1.63)	0.026
	Yes	100 (15.1%)			480 (72.3%)		
**Type of hospital**	Reference university	150 (15.9%)	1.00		622 (66.0%)	1.00	<0.0001***
	Regional county	104 (25.6%)	1.82 (1.37-2.41)	<0.0001	299 (74.0%)	1.47 (1.13-1.90)	0.0040
	Local county	25 (29.8%)	2.24 (1.36-3.69)	0.0016	76 (90.5%)	4.89 (2.33-10.25)	<0.0001
**Type of ward**	Reference: IMU	7 (18.4%)	1.00		26 (68.4%)	1.00	0.0079***
	Postop/ICU	227 (28.0%)	1.72 (0.75-3.97)	0.20	614 (75.8%)	1.45 (0.72-2.92)	0.30
	Surgical ward	42 (8.2%)	0.40 (0.16-0.96)	0.039	348 (68.1%)	0.99 (0.49-2.00)	0.97

Abd: Abdominal, ICU: Intensive Care Unit, IMU: Intermediate Unit, OR: Odds Ratio, Postop: Postoperative Unit. ***) p-value for the entire effect/factor/variable

¤ Acute surgery was defined as surgery within 24 hours after decision of surgery and subacute surgery within a week after the decision.

For peer review only

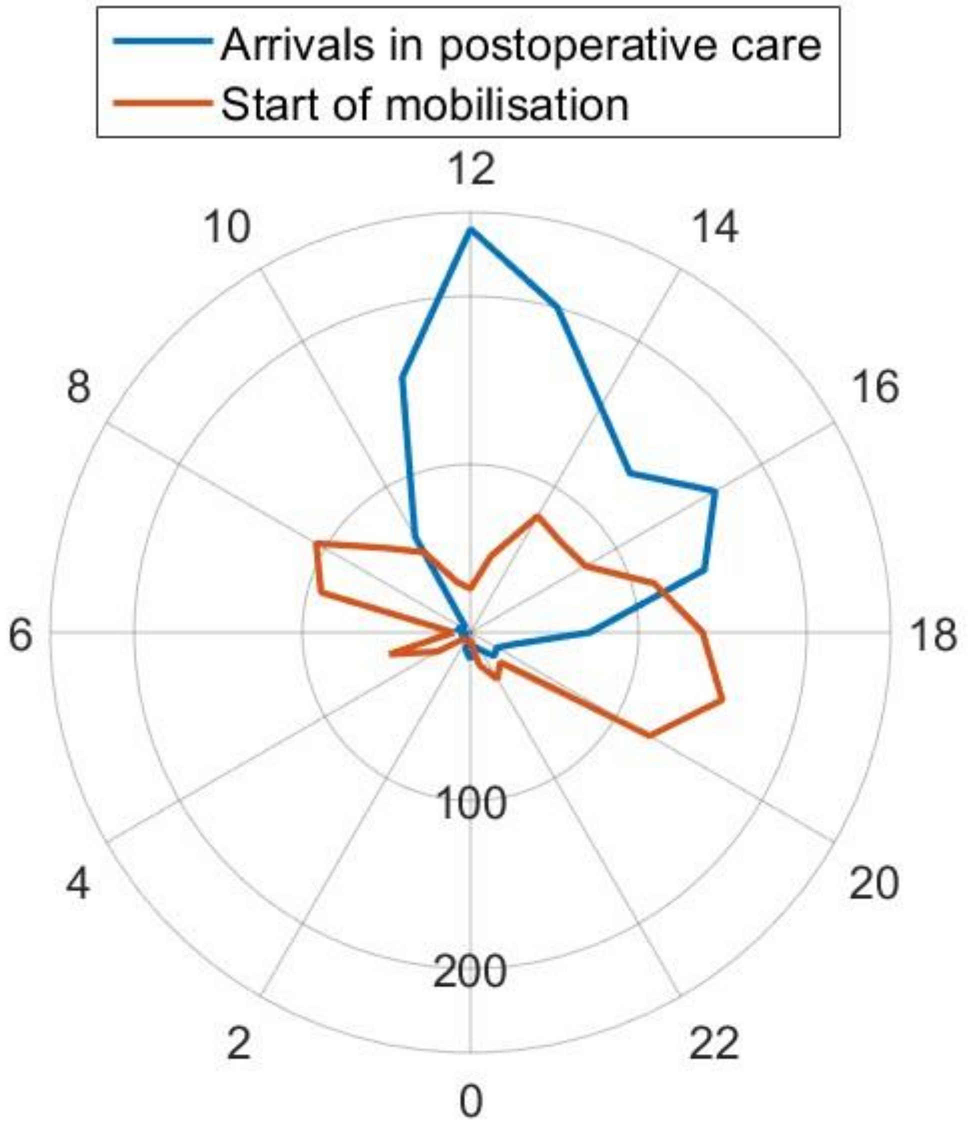

Supplementary Figure 1.

STROBE Statement—Checklist of items that should be included in reports of ***cohort studies***

	Item No	Recommendation	Page No
**Title and abstract**	1	(*a*) Indicate the study's design with a commonly used term in the title or the abstract	Title
		(*b*) Provide in the abstract an informative and balanced summary of what was done and what was found	2
**Introduction**			
Background/rationale	2	Explain the scientific background and rationale for the investigation being reported	4
Objectives	3	State specific objectives, including any prespecified hypotheses	4
**Methods**			
Study design	4	Present key elements of study design early in the paper	5
Setting	5	Describe the setting, locations, and relevant dates, including periods of recruitment, exposure, follow-up, and data collection	5
Participants	6	(*a*) Give the eligibility criteria, and the sources and methods of selection of participants. Describe methods of follow-up	5-6
		(*b*) For matched studies, give matching criteria and number of exposed and unexposed	
Variables	7	Clearly define all outcomes, exposures, predictors, potential confounders, and effect modifiers. Give diagnostic criteria, if applicable	7
Data sources/ measurement	8*	For each variable of interest, give sources of data and details of methods of assessment (measurement). Describe comparability of assessment methods if there is more than one group	7
Bias	9	Describe any efforts to address potential sources of bias	5-7
Study size	10	Explain how the study size was arrived at	8 Fig1
Quantitative variables	11	Explain how quantitative variables were handled in the analyses. If applicable, describe which groupings were chosen and why	7-8
Statistical methods	12	(*a*) Describe all statistical methods, including those used to control for confounding	7-8
		(*b*) Describe any methods used to examine subgroups and interactions	
		(*c*) Explain how missing data were addressed	
		(*d*) If applicable, explain how loss to follow-up was addressed	
		(*e*) Describe any sensitivity analyses	
**Results**			
Participants	13*	(a) Report numbers of individuals at each stage of study—eg numbers potentially eligible, examined for eligibility, confirmed eligible, included in the study, completing follow-up, and analysed	8 Fig1
		(b) Give reasons for non-participation at each stage	
		(c) Consider use of a flow diagram	
Descriptive data	14*	(a) Give characteristics of study participants (eg demographic, clinical, social) and information on exposures and potential confounders	8 Table1
		(b) Indicate number of participants with missing data for each variable of interest	
		(c) Summarise follow-up time (eg, average and total amount)	
Outcome data	15*	Report numbers of outcome events or summary measures over time	9-10 Table2

For peer review only - http://bmjopen.bmj.com/site/about/guidelines.xhtml

Main results	16	(*a*) Give unadjusted estimates and, if applicable, confounder-adjusted estimates and their precision (eg, 95% confidence interval). Make clear which confounders were adjusted for and why they were included<br><br>(*b*) Report category boundaries when continuous variables were categorized<br>(*c*) If relevant, consider translating estimates of relative risk into absolute risk for a meaningful time period	9-10 Table2,3 Supplementary table Fig2,3
Other analyses	17	Report other analyses done—eg analyses of subgroups and interactions, and sensitivity analyses	9-10 Fig2, 3
**Discussion**			
Key results	18	Summarise key results with reference to study objectives	11
Limitations	19	Discuss limitations of the study, taking into account sources of potential bias or imprecision. Discuss both direction and magnitude of any potential bias	12-13
Interpretation	20	Give a cautious overall interpretation of results considering objectives, limitations, multiplicity of analyses, results from similar studies, and other relevant evidence	11-13
Generalisability	21	Discuss the generalisability (external validity) of the study results	13
**Other information**			
Funding	22	Give the source of funding and the role of the funders for the present study and, if applicable, for the original study on which the present article is based	14

*Give information separately for exposed and unexposed groups.

Note: An Explanation and Elaboration article discusses each checklist item and gives methodological background and published examples of transparent reporting. The STROBE checklist is best used in conjunction with this article (freely available on the Web sites of PLoS Medicine at http://www.plosmedicine.org/, Annals of Internal Medicine at http://www.annals.org/, and Epidemiology at http://www.epidem.com/). Information on the STROBE Initiative is available at http://www.strobe-statement.org.

For peer review only
